# Supplementary material for: The Fitness Effects of Codon Composition of the Horizontally Transferred Antibiotic Resistance Genes Intensify at Sub-lethal Antibiotic Levels
Source: Mol Biol Evol. 2023 May 23;40(6):msad123. doi: 10.1093/molbev/msad123 (PMC10246835; doi:10.1093/molbev/msad123)
Supplement: msad123_Supplementary_Data [file msad123_supplementary_data.zip › table S8.pdf]

Supplementary Table 6- primers used in the study

Primer number	Primer name	Sequence
1	qPCR folA Fwd	ATC GGG AAA TCT CAT ATG
2	qPCR folA Rev	GTG ATG GTG ATG GTG AG
3	16s Fwd	GGT TAC CTT GTT ACG ACT T
4	16s Rev	CGG TGA ATA CGT TCY CGG
5	*N.sicca* First15 Fwd Primer	GGC ATC AAC AAC ACC ATG CCG TGG CAT CTG CCG GAA GAT TTC GCG TTC TTC
6	*N.sicca* First15 Rev Primer	AGA TGC CAC GGC ATG GTG TTG TTG ATG CCK ATG CAG CGR TCS GGC GCG CAS GCS GCR ATC AGG GTG ATT TTC GGC ATA TG
7	*N.sicca* Second15 Fwd Primer	TTC GCG TTC TTC AAA AGC TAC ACC CTG GAT AAA CCG GTG GTG ATG GGC CGC AAA ACC TGG G
8	*N.sicca* Second15 Rev Primer	ATC CAG GGT GTA GCT TTT GAA GAA CGC GAA RTC YTC SGG CAR RTG CCA MGG CAT GGT GTT GTT GAT GCC G
9	*L.grayi* First15 Fwd Primer	GGC AAC ATC GGC AAA GAT AAC CAA ATG CCG TGG CGC CTG CCG GCG GAT CTG G
10	*L.grayi* First15 Rev Primer	CGG CAT TTG GTT ATC TTT GCC GAT GTT GCC RTT CGC ATC TTG SGC CCA RAT CAR RTT GAT CAT ATG AGA TTT CCC G
11	*L.grayi* Second15 Fwd Primer	CGA ACG GCA ACA TCG GCA AAG AYA ACC AAA TGC CKT GGC GMC TGC CRG CKG ATC TGG CKT ACT TCA AAA AAC AAA CCA CC
12	*L.grayi* Second15 Rev Primer	TTT GCC GAT GTT GCC GTT CGC ATC TTG CGC CCA GAT CAG GTT GAT C
13	*E.coli* First15 Fwd Primer	ATC GGG AAA TCT CAT ATG ATC AGY CTG ATY GCG GCG YTR GCG GTR GAT CGC GTK ATC GGC ATG GAA AAC GCG ATG C
14	*E.coli* First15 Rev Primer	CTG ATC ATA TGA GAT TTC CCG ATA AAA AAA ATT GTC GCC ACT ATA CGT AAA GCG TAA ACC GTC GTC GAC TGG TGC GAG G
15	*E.coli* Second15 Fwd Primer	TTG TTC AGG GTG TTG CGT TTG AAC CAS GCS AGA TCS GCM GGC AGG TTC CAC GGC ATS GCG TTT TCC ATG CCG ATC ACG CG
16	*E.coli* Second15 Rev Primer	TGG TTC AAA CGC AAC ACC CTG AAC AAA CCG GTG ATC ATG GGC CGC CAT ACC TGG GAA AGC ATC GG
17	Genome recombination Fwd	GAA GAA GGT AAA CAT ACC GGC AAC ATG GCG GAT GAA CCG GAA ACG AAA CCC TCA TCC TAA TCA TGA TCA TCG CAG TAC TGT TG
18	Genome recombination Rev	AAG GCC GGA TAA GAC GCG ACC GGC GTC GCA TCC GGC GCT AGC CGT AAA TTC TAT ACA AAA CTG TCA AAC

19	FWD 2 Deep seq	TCG TCG GCA GCG TCA GAT GTG TAT AAG AGA CAG NNN NNN NTC CTC AAC ATC ATC CTC GCA CCA GTC GAC G
20	Rev Deep seq	GTC TCG TGG GCT CGG AGA TGT GTA TAA GAG ACA GNN NNN NNG AAC TTC GAA GCA GCT CCA GCC TAC
21	adkFWD	ATG CGT ATC ATT CTG CTT GG
22	adkREV	TTT GCT TGT TTA CCC AGC TC
