# Supplementary material for: The Fitness Effects of Codon Composition of the Horizontally Transferred Antibiotic Resistance Genes Intensify at Sub-lethal Antibiotic Levels
Source: Mol Biol Evol. 2023 May 23;40(6):msad123. doi: 10.1093/molbev/msad123 (PMC10246835; doi:10.1093/molbev/msad123)
Supplement: msad123_Supplementary_Data [file msad123_supplementary_data.zip › table S9.pdf]

Supplementary Table 7: Nextera Index primers used in the deep sequencing experiment

	DHFR Type	index primer 1	index primer 2	index primer 1	index primer 2	Library	TMP [µgr/ml]	Day	Remarks
1	L.grayi	S517	N1	GCGTAAGA	TCGCCTTA	15.1	no	0	Day0 population
2	L.grayi	S517	N2	GCGTAAGA	CTAGTACG	15.1	no	1	
3	L.grayi	S517	N3	GCGTAAGA	TTCTGCCT	15.1	no	2	
4	L.grayi	S517	N4	GCGTAAGA	GCTCAGGA	15.1	0.03	1	
5	L.grayi	S517	N5	GCGTAAGA	AGGAGTCC	15.1	0.03	2	
6	L.grayi	S517	N6	GCGTAAGA	CATGCCTA	15.1	0.1	1	
7	L.grayi	S517	N7	GCGTAAGA	GTAGAGAG	15.1	0.1	2	
8	L.grayi	S517	N8	GCGTAAGA	CCTCTCTG	15.1	0.3	1	
9	L.grayi	S517	N9	GCGTAAGA	AGCGTAGC	15.1	0.3	2	
10	L.grayi	S517	N10	GCGTAAGA	CAGCCTCG	15.2	no	0	Day0 population
11	L.grayi	S517	N11	GCGTAAGA	TGCCTCTT	15.2	no	1	
12	L.grayi	S517	N12	GCGTAAGA	TCCTCTAC	15.2	no	2	
13	L.grayi	S502	N1	CTCTCTAT	TCGCCTTA	15.2	0.03	1	
14	L.grayi	S502	N2	CTCTCTAT	CTAGTACG	15.2	0.03	2	
15	L.grayi	S502	N3	CTCTCTAT	TTCTGCCT	15.2	0.1	1	
16	L.grayi	S502	N4	CTCTCTAT	GCTCAGGA	15.2	0.1	2	
17	L.grayi	S502	N5	CTCTCTAT	AGGAGTCC	15.2	0.3	1	
18	L.grayi	S502	N6	CTCTCTAT	CATGCCTA	15.2	0.3	2	
28	N.sicca	S503	N4	TATCCTCT	GCTCAGGA	15.1	no	0	Day0 population
29	N.sicca	S503	N5	TATCCTCT	AGGAGTCC	15.1	no	1	
30	N.sicca	S503	N6	TATCCTCT	CATGCCTA	15.1	no	2	
31	N.sicca	S503	N7	TATCCTCT	GTAGAGAG	15.1	20	1	
32	N.sicca	S503	N8	TATCCTCT	CCTCTCTG	15.1	20	2	
33	N.sicca	S503	N9	TATCCTCT	AGCGTAGC	15.1	100	1	
34	N.sicca	S503	N10	TATCCTCT	CAGCCTCG	15.1	100	2	
35	N.sicca	S503	N11	TATCCTCT	TGCCTCTT	15.1	200	1	
36	N.sicca	S503	N12	TATCCTCT	TCCTCTAC	15.1	200	2	
37	N.sicca	S504	N1	AGAGTAGA	TCGCCTTA	15.2	no	0	Day0 population
38	N.sicca	S504	N2	AGAGTAGA	CTAGTACG	15.2	no	1	
39	N.sicca	S504	N3	AGAGTAGA	TTCTGCCT	15.2	no	2	
40	N.sicca	S504	N4	AGAGTAGA	GCTCAGGA	15.2	20	1	
41	N.sicca	S504	N5	AGAGTAGA	AGGAGTCC	15.2	20	2	
42	N.sicca	S504	N6	AGAGTAGA	CATGCCTA	15.2	100	1	
43	N.sicca	S504	N7	AGAGTAGA	GTAGAGAG	15.2	100	2	
44	N.sicca	S504	N8	AGAGTAGA	CCTCTCTG	15.2	200	1	
45	N.sicca	S504	N9	AGAGTAGA	AGCGTAGC	15.2	200	2	
55	E.coli	S505	N7	GTAAGGAG	GTAGAGAG	15.1	no	0	Day0 population

56	E.coli	S505	N8	GTAAGGAG	CCTCTCTG	15.1	no	1	
57	E.coli	S505	N9	GTAAGGAG	AGCGTAGC	15.1	no	2	
58	E.coli	S505	N10	GTAAGGAG	CAGCCTCG	15.1	0.03	1	
59	E.coli	S505	N11	GTAAGGAG	TGCCTCTT	15.1	0.03	2	
62	E.coli	S506	N2	ACTGCATA	CTAGTACG	15.1	0.3	1	
63	E.coli	S506	N3	ACTGCATA	TTCTGCCT	15.1	0.3	2	
64	E.coli	S506	N4	ACTGCATA	GCTCAGGA	15.2	no	0	Day0 population
65	E.coli	S506	N5	ACTGCATA	AGGAGTCC	15.2	no	1	
66	E.coli	S506	N6	ACTGCATA	CATGCCTA	15.2	no	2	
67	E.coli	S506	N7	ACTGCATA	GTAGAGAG	15.2	0.03	1	
68	E.coli	S506	N8	ACTGCATA	CCTCTCTG	15.2	0.03	2	
71	E.coli	S506	N11	ACTGCATA	TGCCTCTT	15.2	0.3	1	
72	E.coli	S506	N12	ACTGCATA	TCCTCTAC	15.2	0.3	2	

	DHFR Type	index primer 1	index primer 2	index primer 1	index primer 2	Library	TMP [ugr/ml]	Day	Remarks
1	L.grayi	S517	N1	GCGTAAGA	TCGCCTTA	15.1	no	0	Day0 population
2	L.grayi	S517	N2	GCGTAAGA	CTAGTACG	15.1	no	1	
3	L.grayi	S517	N3	GCGTAAGA	TTCTGCCT	15.1	no	2	
4	L.grayi	S517	N4	GCGTAAGA	GCTCAGGA	15.1	0.03	1	
5	L.grayi	S517	N5	GCGTAAGA	AGGAGTCC	15.1	0.03	2	
6	L.grayi	S517	N6	GCGTAAGA	CATGCCTA	15.1	0.1	1	
7	L.grayi	S517	N7	GCGTAAGA	GTAGAGAG	15.1	0.1	2	
8	L.grayi	S517	N8	GCGTAAGA	CCTCTCTG	15.1	0.3	1	
9	L.grayi	S517	N9	GCGTAAGA	AGCGTAGC	15.1	0.3	2	
10	L.grayi	S517	N10	GCGTAAGA	CAGCCTCG	15.2	no	0	Day0 population
11	L.grayi	S517	N11	GCGTAAGA	TGCCTCTT	15.2	no	1	
12	L.grayi	S517	N12	GCGTAAGA	TCCTCTAC	15.2	no	2	
13	L.grayi	S502	N1	CTCTCTAT	TCGCCTTA	15.2	0.03	1	
14	L.grayi	S502	N2	CTCTCTAT	CTAGTACG	15.2	0.03	2	
15	L.grayi	S502	N3	CTCTCTAT	TTCTGCCT	15.2	0.1	1	
16	L.grayi	S502	N4	CTCTCTAT	GCTCAGGA	15.2	0.1	2	
17	L.grayi	S502	N5	CTCTCTAT	AGGAGTCC	15.2	0.3	1	
18	L.grayi	S502	N6	CTCTCTAT	CATGCCTA	15.2	0.3	2	
28	N.sicca	S503	N4	TATCCTCT	GCTCAGGA	15.1	no	0	Day0 population
29	N.sicca	S503	N5	TATCCTCT	AGGAGTCC	15.1	no	1	
30	N.sicca	S503	N6	TATCCTCT	CATGCCTA	15.1	no	2	
31	N.sicca	S503	N7	TATCCTCT	GTAGAGAG	15.1	20	1	
32	N.sicca	S503	N8	TATCCTCT	CCTCTCTG	15.1	20	2	
33	N.sicca	S503	N9	TATCCTCT	AGCGTAGC	15.1	100	1	
34	N.sicca	S503	N10	TATCCTCT	CAGCCTCG	15.1	100	2	
35	N.sicca	S503	N11	TATCCTCT	TGCCTCTT	15.1	200	1	
36	N.sicca	S503	N12	TATCCTCT	TCCTCTAC	15.1	200	2	
37	N.sicca	S504	N1	AGAGTAGA	TCGCCTTA	15.2	no	0	Day0 population
38	N.sicca	S504	N2	AGAGTAGA	CTAGTACG	15.2	no	1	
39	N.sicca	S504	N3	AGAGTAGA	TTCTGCCT	15.2	no	2	
40	N.sicca	S504	N4	AGAGTAGA	GCTCAGGA	15.2	20	1	
41	N.sicca	S504	N5	AGAGTAGA	AGGAGTCC	15.2	20	2	
42	N.sicca	S504	N6	AGAGTAGA	CATGCCTA	15.2	100	1	
43	N.sicca	S504	N7	AGAGTAGA	GTAGAGAG	15.2	100	2	
44	N.sicca	S504	N8	AGAGTAGA	CCTCTCTG	15.2	200	1	
45	N.sicca	S504	N9	AGAGTAGA	AGCGTAGC	15.2	200	2	
55	E.coli	S505	N7	GTAAGGAG	GTAGAGAG	15.1	no	0	Day0 population
56	E.coli	S505	N8	GTAAGGAG	CCTCTCTG	15.1	no	1	
57	E.coli	S505	N9	GTAAGGAG	AGCGTAGC	15.1	no	2	

58	E.coli	S505	N10	GTAAGGAG	CAGCCTCG	15.1	0.03	1	
59	E.coli	S505	N11	GTAAGGAG	TGCCTCTT	15.1	0.03	2	
60	E.coli	S505	N12	GTAAGGAG	TCCTCTAC	15.1	0.1	1	
61	E.coli	S506	N1	ACTGCATA	TCGCCTTA	15.1	0.1	2	
62	E.coli	S506	N2	ACTGCATA	CTAGTACG	15.1	0.3	1	
63	E.coli	S506	N3	ACTGCATA	TTCTGCCT	15.1	0.3	2	
64	E.coli	S506	N4	ACTGCATA	GCTCAGGA	15.2	no	0	Day0 population
65	E.coli	S506	N5	ACTGCATA	AGGAGTCC	15.2	no	1	
66	E.coli	S506	N6	ACTGCATA	CATGCCTA	15.2	no	2	
67	E.coli	S506	N7	ACTGCATA	GTAGAGAG	15.2	0.03	1	
68	E.coli	S506	N8	ACTGCATA	CCTCTCTG	15.2	0.03	2	
69	E.coli	S506	N9	ACTGCATA	AGCGTAGC	15.2	0.1	1	
70	E.coli	S506	N10	ACTGCATA	CAGCCTCG	15.2	0.1	2	
71	E.coli	S506	N11	ACTGCATA	TGCCTCTT	15.2	0.3	1	
72	E.coli	S506	N12	ACTGCATA	TCCTCTAC	15.2	0.3	2	
