# Supplementary material for: The Fitness Effects of Codon Composition of the Horizontally Transferred Antibiotic Resistance Genes Intensify at Sub-lethal Antibiotic Levels
Source: Mol Biol Evol. 2023 May 23;40(6):msad123. doi: 10.1093/molbev/msad123 (PMC10246835; doi:10.1093/molbev/msad123)
Supplement: msad123_Supplementary_Data [file msad123_supplementary_data.zip › MBE_Suppl. Figures_R1.pdf]

# Supplementary Figures

## The fitness effects of codon composition of the horizontally transferred dihydrofolate reductase genes intensify at sub-lethal trimethoprim levels.

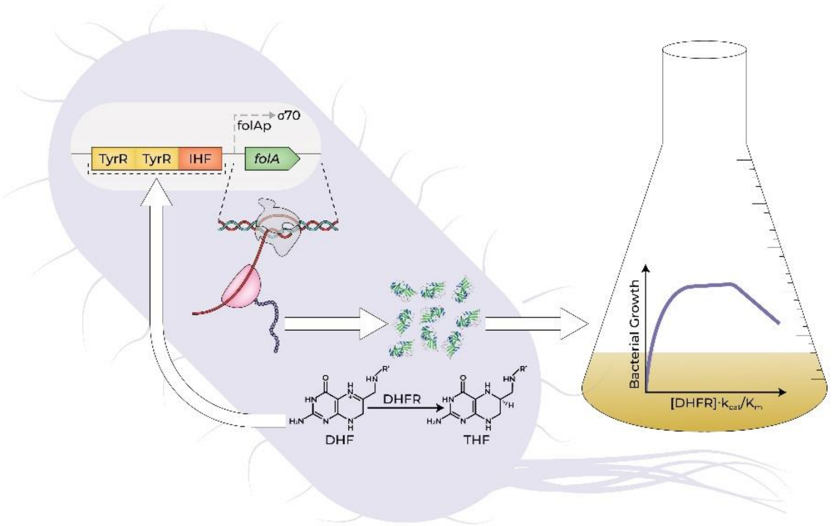


**Fig S1.** Relationship between *folA* promoter activity, functional capacity of DHFR, and bacterial growth. DHFR catalyzes the formation of tetrahydrofolate (THF), a one-carbon carrier, from dihydrofolate (DHF). Drop in functional capacity of DHFR (a product of the intracellular DHFR levels, [DHFR], and the catalytic proficiency of DHFR, $k_{cat}/K_M$) due to mutations in *folA* (Bershtein, Choi, et al. 2015) or TMP treatment (Bollenbach, et al. 2009; Bershtein, Choi, et al. 2015) leads to an upregulation of *folA* promoter (*folAp*) via a metabolic regulation loop operating through binding of TyrR transcription activator to TyrR boxes (Yang, et al. 2007). The decrease in bacterial growth due to a drop in DHFR functional capacity follows a Michaelis-Menten-like dynamics (Bershtein, Serohijos, et al. 2015; Rodrigues, et al. 2016). The increase in DHFR abundance above a basal level is also accompanied by a drop in bacterial growth (Bhattacharyya, et al. 2016). IHF, a binding site to Integration Host Factor (Yang, et al. 2007). σ70, initiation of transcription by sigma factor 70 of RNA polymerase.

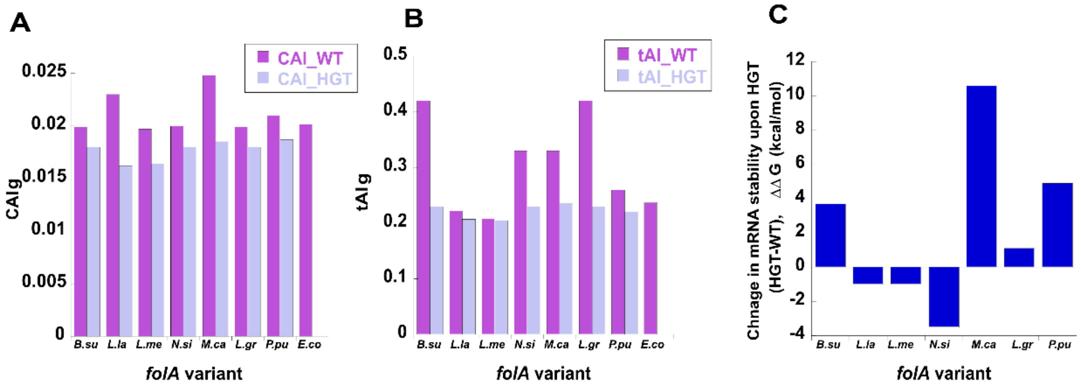


**Fig S2.** The effect of HGT on codon optimality and 5'-end mRNA stability of the transferred genes. *folA* variants are marked as in **table 1.** (*A*,*B*) Change in the (*A*) CAIg and (*B*) tAIg values of *folA* genes upon transfer from the original organism (CAI_WT) to *E. coli* (CAI_HGT). (*C*) Change in mRNA folding stability (ΔΔG, kcal/mol) of 30-nt long 5'-end mRNA structure (from nucleotide -25 of the upstream sequence and up to nucleotide +5 within the coding sequence), calculated by subtraction of ΔG in *E. coli* (HGT) from that in the original organism (WT).

A

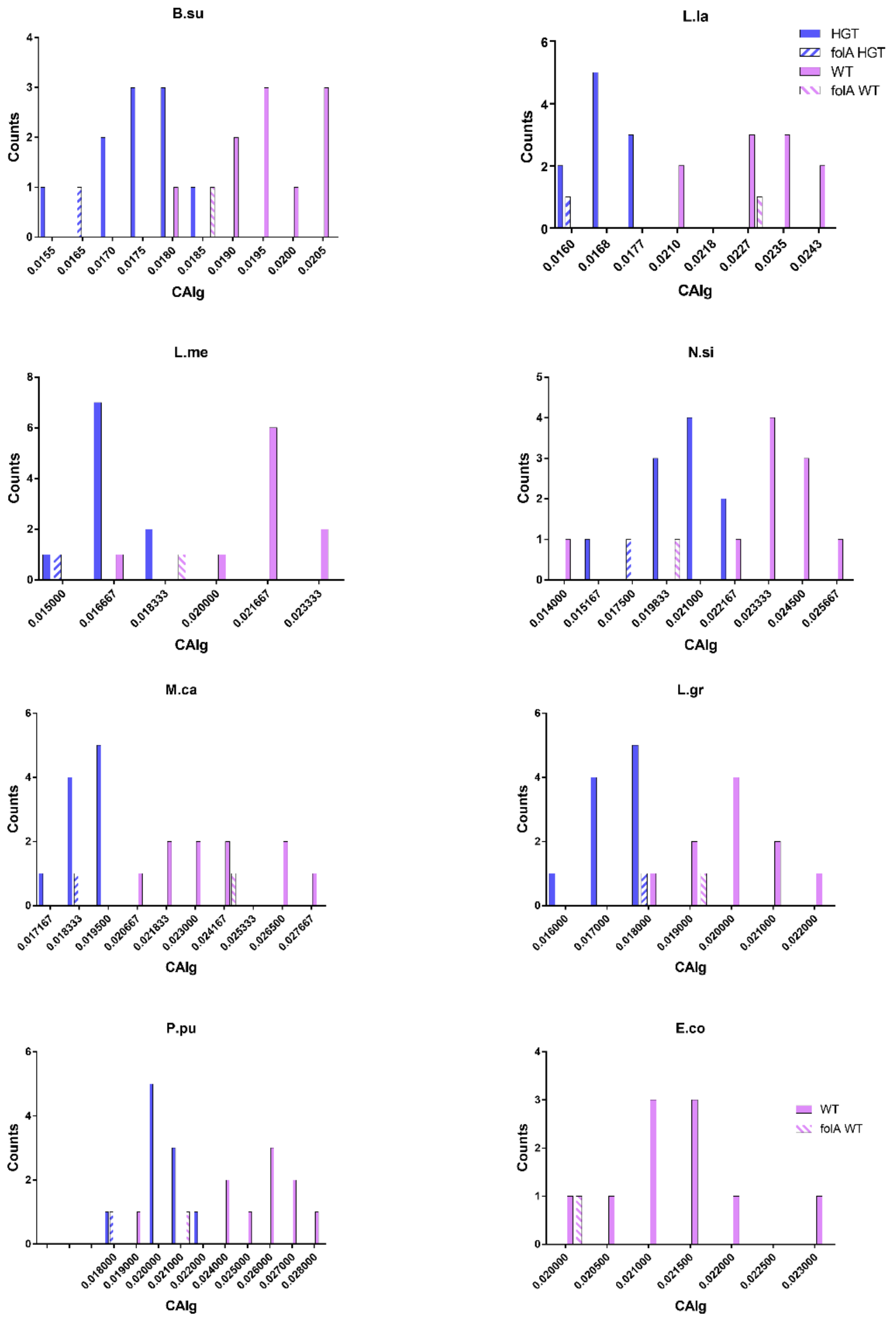
B.su
L.la
HGT
folA HGT
WT
folA WT
L.me
N.si
M.ca
L.gr
P.pu
E.co
WT
folA WT
Counts
CAIg

B

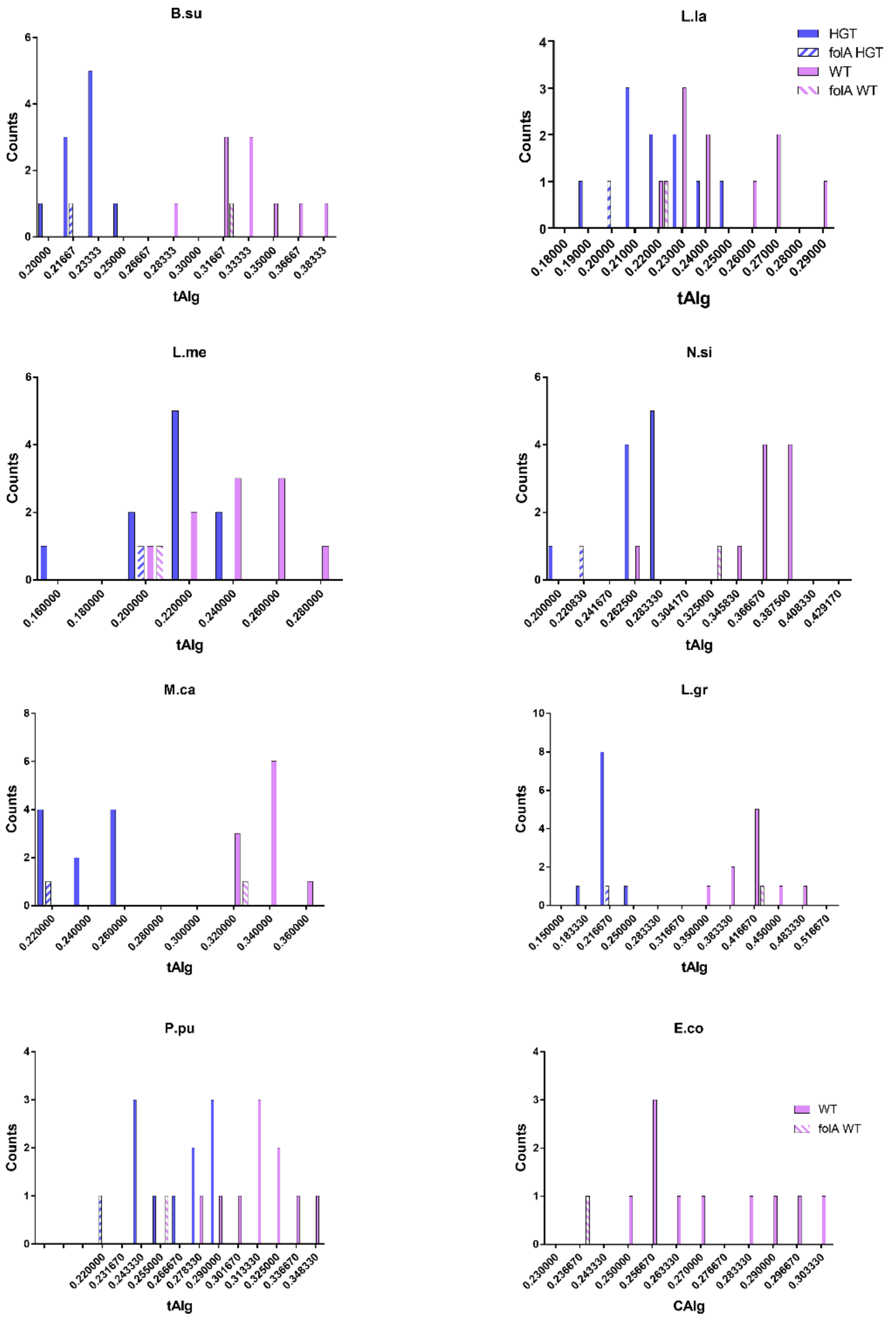
B.su
L.la
L.me
N.si
M.ca
L.gr
P.pu
E.co
Counts
tAlg
CAlg
HGT
folA HGT
WT
folA WT

C

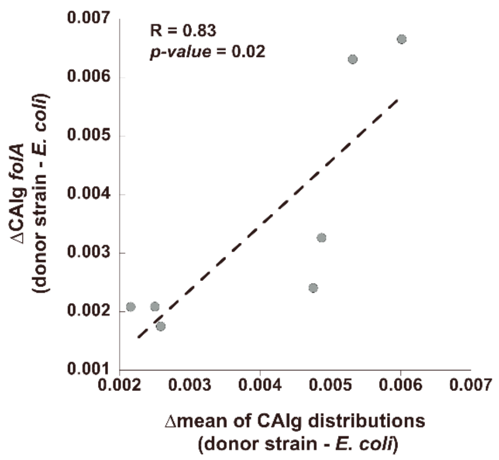
R = 0.83
p-value = 0.02
ΔCAlg folA
(donor strain - E. coli)
Δmean of CAlg distributions
(donor strain - E. coli)
0.001
0.002
0.003
0.004
0.005
0.006
0.007

D

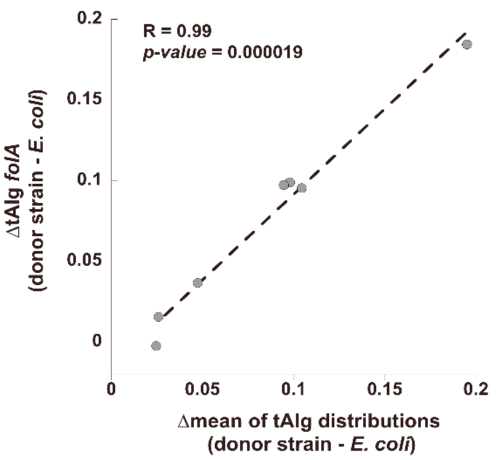
R = 0.99
p-value = 0.000019
ΔtAlg folA
(donor strain - E. coli)
Δmean of tAlg distributions
(donor strain - E. coli)
0
0.05
0.1
0.15
0.2

**Fig S3.** Distribution of (*A*) CAIg and (*B*) tAIg values of ten orthologous genes encoding central metabolic genes in the donor bacteria (*aroK, cysE, pgk, glnA, zwf, gdhA, leuA, metE, adk, metK*). The data are binned and presented as histograms. WT (*pink*), values are calculated according to the CAIg and tAIg metrics of the donor bacteria (bacterial annotations are listed in **table 1**). HGT (blue), CAIg and tAIg values calculated according to *E. coli*'s metrics. Dashed bars, CAIg and tAIg values of *folA* gene. (*C, D*) Correlations between the difference in means of distributions (Δmean) of CAIg (*C*) or tAIg (*D*) values obtained by subtracting the values obtained with the metrics of each donor species from that of *E. coli* and difference in CAIg or tAIg values of *folA* gene obtained from the corresponding metrics.

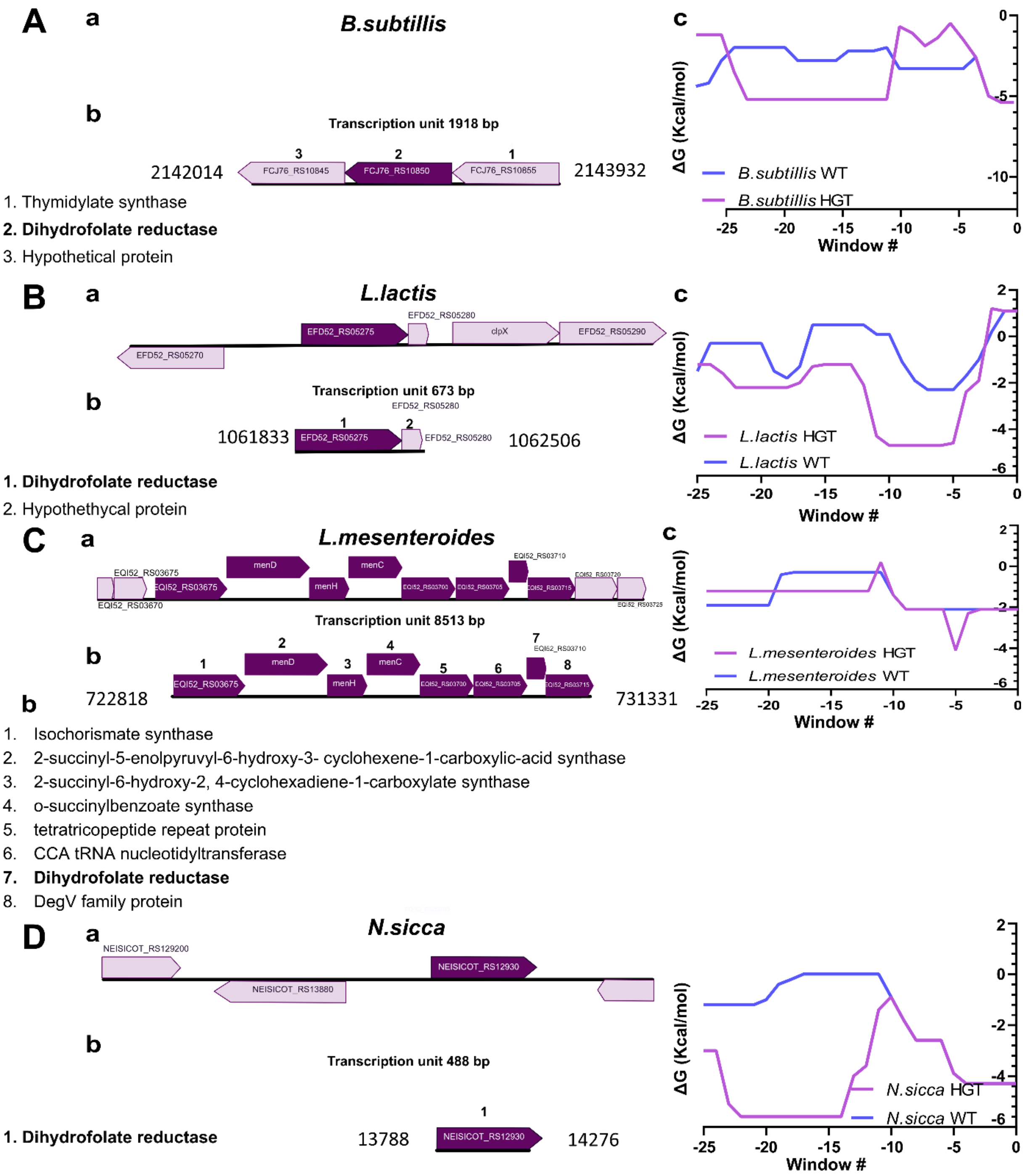
A
a
B.subtillis
b
Transcription unit 1918 bp
3
2
1
2142014
FCJ76_RS10845
FCJ76_RS10850
FCJ76_RS10855
2143932
1. Thymidylate synthase
2. Dihydrofolate reductase
3. Hypothetical protein
c
ΔG (Kcal/mol)
B.subtillis WT
B.subtillis HGT
Window #
B
a
L.lactis
EFD52_RS05275
EFD52_RS05280
clpX
EFD52_RS05290
EFD52_RS05270
b
Transcription unit 673 bp
1
2
1061833
EFD52_RS05275
EFD52_RS05280
1062506
1. Dihydrofolate reductase
2. Hypothethycal protein
c
ΔG (Kcal/mol)
L.lactis HGT
L.lactis WT
Window #
C
a
L.mesenteroides
menD
menC
menH
EQI52_RS03675
EQI52_RS03670
EQI52_RS03710
Transcription unit 8513 bp
b
1
2
3
4
5
6
7
8
722818
731331
1. Isochorismate synthase
2. 2-succinyl-5-enolpyruvyl-6-hydroxy-3- cyclohexene-1-carboxylic-acid synthase
3. 2-succinyl-6-hydroxy-2, 4-cyclohexadiene-1-carboxylate synthase
4. o-succinylbenzoate synthase
5. tetratricopeptide repeat protein
6. CCA tRNA nucleotidyltransferase
7. Dihydrofolate reductase
8. DegV family protein
c
ΔG (Kcal/mol)
L.mesenteroides HGT
L.mesenteroides WT
Window #
D
a
N.sicca
NEISICOT_RS129200
NEISICOT_RS12930
NEISICOT_RS13880
b
Transcription unit 488 bp
1
1. Dihydrofolate reductase
13788
NEISICOT_RS12930
14276
ΔG (Kcal/mol)
N.sicca HGT
N.sicca WT
Window #

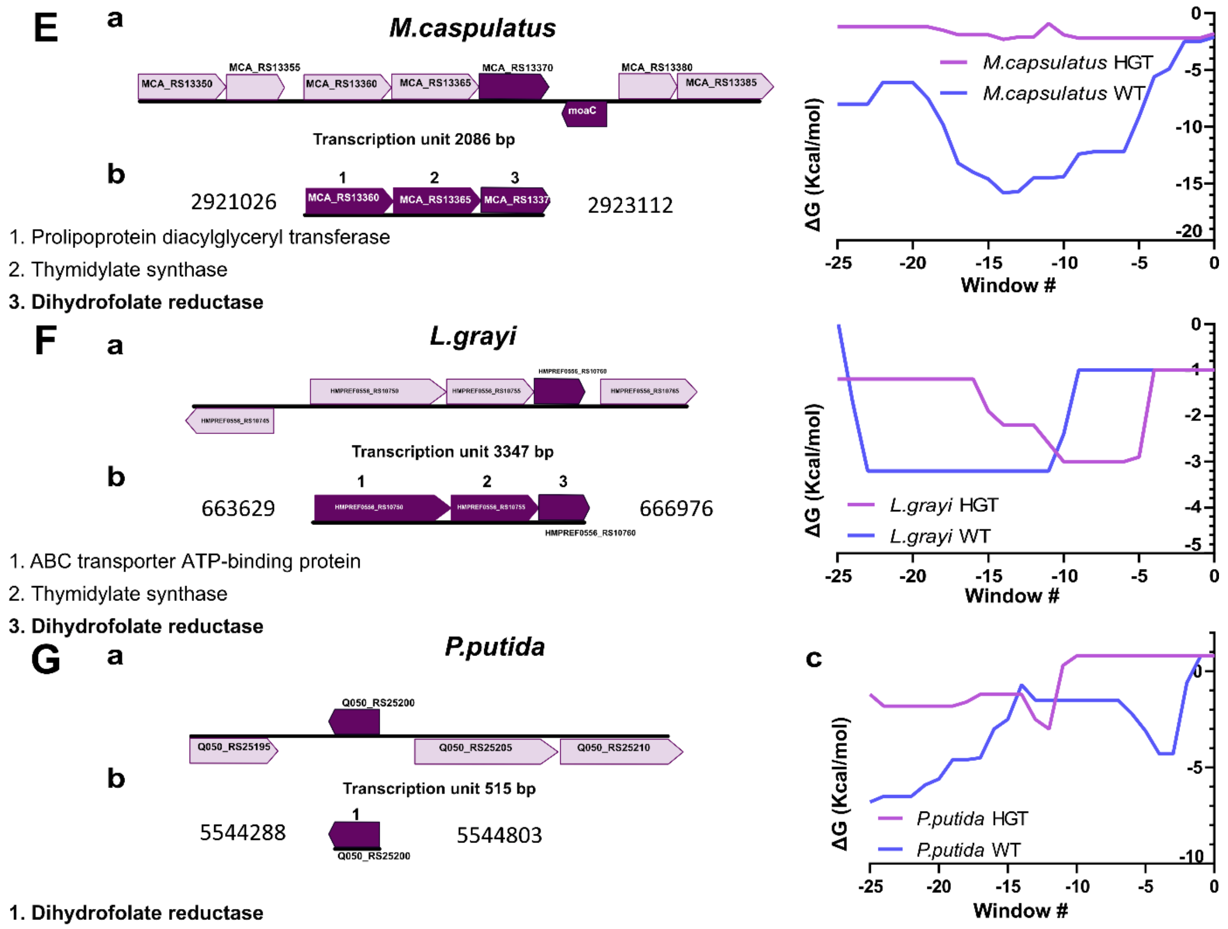


**Fig S4.** Genomic content/transcript composition of the transferred *folA* genes within the original organisms and change in 5'-end mRNA stability upon HGT to *E. coli*. **(***A-G***)** transferred *folA* genes. *Left panels*. (*a*) genomic content and (*b*) predicted compostion and length of mRNA transcripts. *Right panels*. mRNA folding stability (*D*G, kcal/mol) of transferred *folA* genes in their original transcript content (WT) and upon transfer downstream to *E. coli*'s *folA* endogenous promoter (HGT), calculated using a 30 nucleotide-long sliding window starting at -25 nt upstream to the translation start codon and moving 1 nt at a time for a total of 25 windows.

A

| Amino Acid | codon | frequency | codon | frequency | codon | frequency | codon | frequency | codon | frequency | most frequent codon |
|---|---|---|---|---|---|---|---|---|---|---|---|
| A | GCA | 1 | GCC | 4 | **GCG** | **7** | GCT | 1 | | | GCG |
| C | **TGC** | **1** | TGT | 1 | | | | | | | TGC |
| D | GAC | 5 | **GAT** | **8** | | | | | | | GAT |
| E | **GAA** | **9** | GAG | 3 | | | | | | | GAA |
| F | **TTC** | **4** | TTT | 2 | | | | | | | TTC |
| G | GGA | 1 | **GGC** | **5** | GGT | 4 | | | | | GGC |
| H | CAC | 2 | **CAT** | **3** | | | | | | | CAT |
| I | **ATC** | **7** | ATT | 5 | | | | | | | ATC |
| K | **AAA** | **5** | AAG | 1 | | | | | | | AAA |
| L | CTC | 2 | **CTG** | **5** | TTA | 2 | TTG | 2 | | | CTG |
| M | **ATG** | **5** | | | | | | | | | ATG |
| N | **AAC** | **4** | AAT | 2 | | | | | | | AAC |
| P | CCA | 3 | CCC | 1 | **CCG** | **5** | CCT | 1 | | | CCG |
| Q | **CAA** | **2** | CAG | 2 | | | | | | | CAA |
| R | **CGC** | **6** | CGG | 2 | CGT | 1 | | | | | CGC |
| S | **AGC** | **3** | AGT | 2 | TCA | 1 | TCG | 2 | TCT | 1 | AGC |
| T | **ACC** | **3** | ACG | 3 | | | | | | | ACC |
| V | GTA | 4 | **GTG** | **5** | GTT | 2 | | | | | GTG |
| W | **TCG** | **5** | | | | | | | | | TGG |
| Y | **TAC** | **1** | | | | | | | | | TAC |
| STOP | **TAA** | **1** | | | | | | | | | TAA |

B C

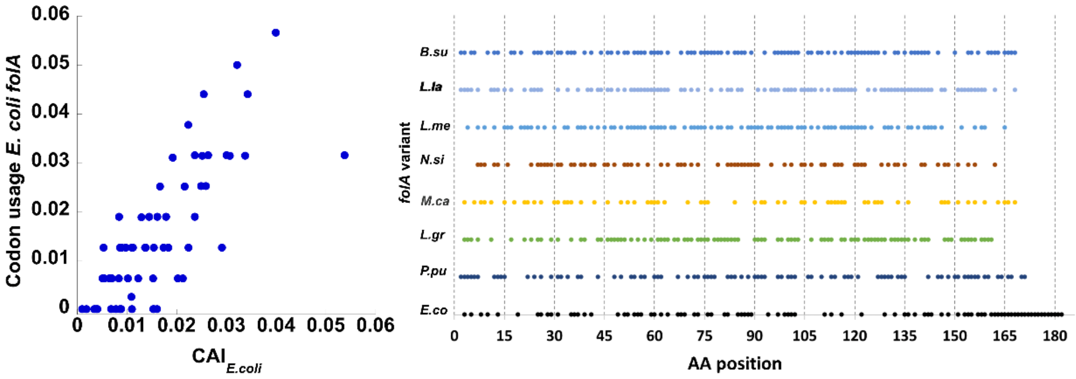


**Fig S5.** Identification of the most frequent codons in *E. coli*'s *folA* coding sequence. (*A*) Total counts of codons comprising *E. coli*'s *folA* coding sequence and identification of the most freqeunt ones (red). (*B*) Correlation between the frequencies of each of the codons comprising *E. coli*'s *folA* coding sequence, $CAI_{E.\ coli}$ (calculated relatively to the codon composition of the *folA* gene) and the corresponding Codon Adaptation Index (CAI) for each codon as determined for *E. coli*.

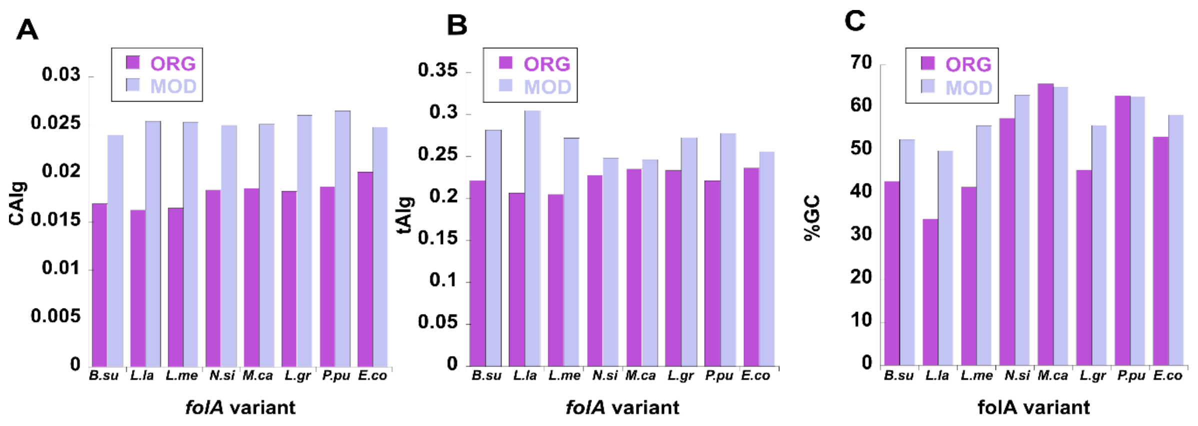


**Fig S6.** Change in codon optimality and GC content upon original to frequent codon replacement within transferred folA genes. *folA* variants are marked as in table 1. (*A*,*B*) Change in the (*A*) CAIg and (*B*) tAIg values of *folA* genes upon replacemnt of the original (ORG) codons to most frequent (MOD) codons (using *E. coli*'s CAI and tAI metrics, **table S2**). (*C*) Change in GC content (%) upon original (ORG) to frequent (MOD) codon replacemnt.

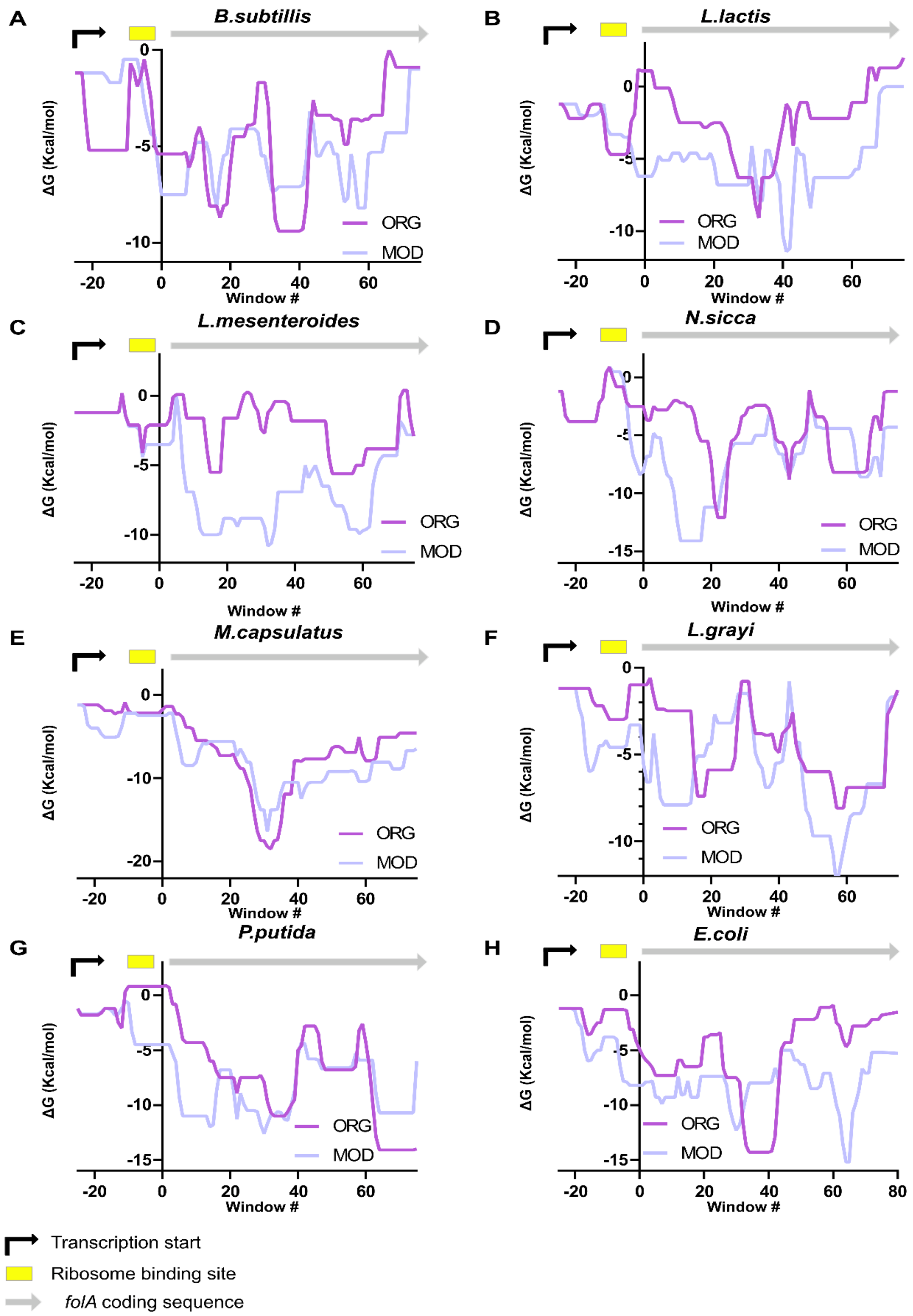


**Fig S7.** mRNA stability profiles of the xenologous *folA* genes. Stability is expressed in Gibbs free energy change between folded and unfolded states of mRNA transcripts (ΔG, kcal/mol) for the MOD and ORG sequences. Stability is calculated with a 30 nt-long sliding window starting from the transcription start (nucleotide -25 upstream to the translation start codon) in steps of 1 nt.

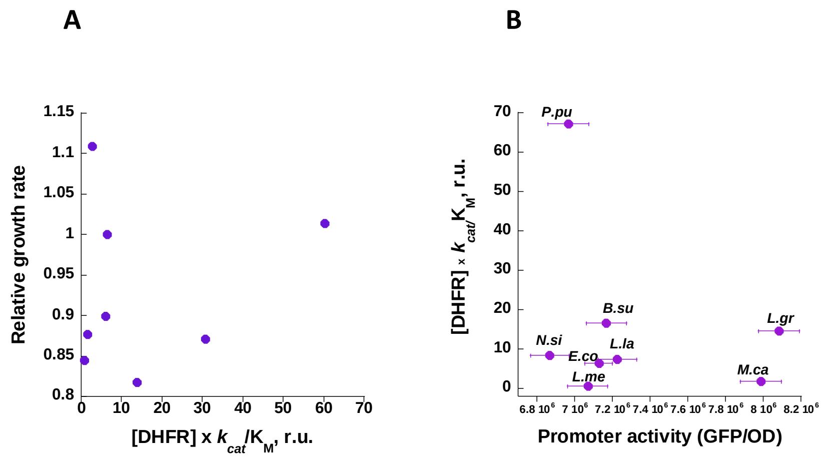


**Fig S8.** DHFR functional capcity is a poor predictor of fitness effects upon HGT of ORG sequences. (*A*) Growth rate of ORG strains as a function of DHFR functional capacity. (*B*) Promoter activity as a function of DHFR functional capacity of ORG strains.

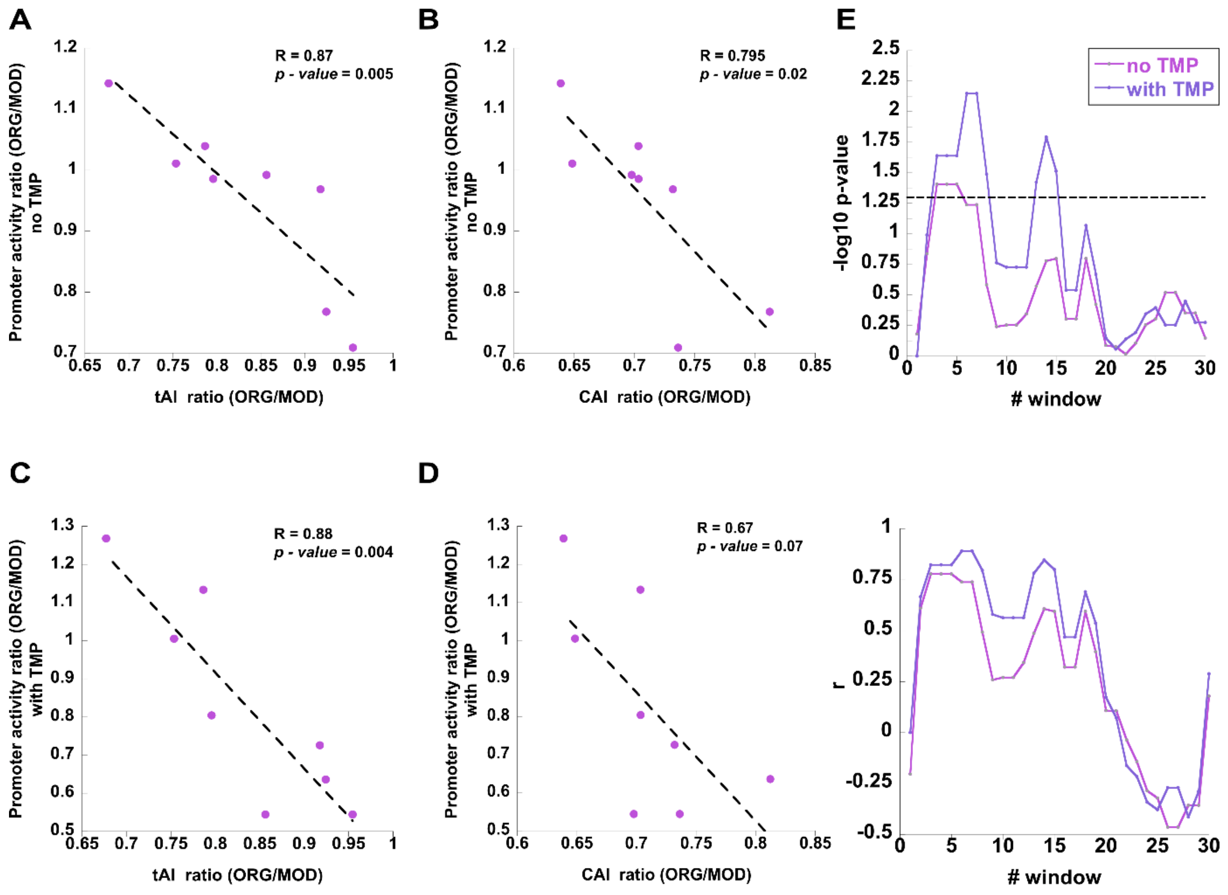


**Fig S9.** Correlation between changes in promoter activity and changes in codon optimality and mRNA stability upon ORG to MOD codon replacemnt in the absense and presence of IC50 TMP levels. (*A*,*B*) Changes in promoter activity (calculated as ORG/MOD ratio) are significantly correlated with changes in tAIg values (calculated as ORG/MOD ratio) in the absence (A) and presence (B) of TMP. **(***C*,*D***)** Correlation between changes in promoter activity and changes in CAIg values in the absence (A) and presence (B) of TMP are not significant, but follow a trend similar to that observed in (*A*,*B*). (*E*) Correlation between the change in promoter activity (calculated as ORG/MOD ratio) and change in mRNA stability (ORG-MOD, ΔΔG, kcal/mol), calculated using a 30 nucleotide-long sliding window starting from the mRNA transcription start (25 nt upstream to the translation start codon) and moving 1 nt at a time for 30 windows, is presented as *p-values* (-log10[p-value]), upper panel, and correlation coefficients, *r*, lower panel, for Spearman non-parametric test. The horizontal bar at the upper panel indicates the significance barrier for $p < 0.05$. *Pink*, no TMP. *Purple*, IC50 levels of TMP.

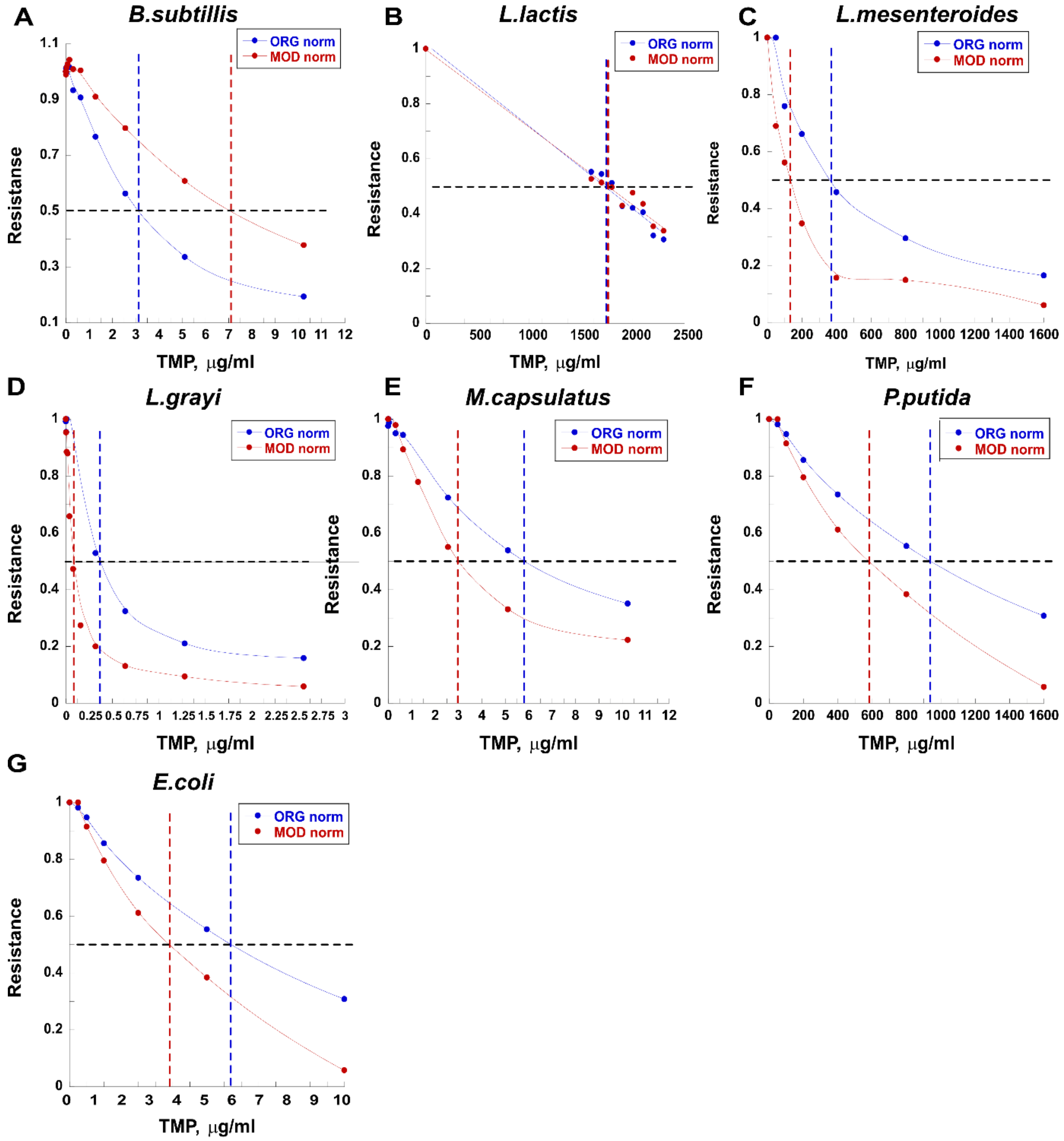


**Fig S10.** Calculation of TMP IC50 levels for *E. coli* strains carrying ORG and MOD *folA* genes. (*A*-*G*) Normalized growth of E. coli strains carrying ORG (blue) or MOD (red) *folA* gene is plotted as a function of TMP concentrations. Dashed line intersections with the black line indicate TMP concentrations at which growth of each variant reaches 50% of its growth in the absence of TMP (IC50).

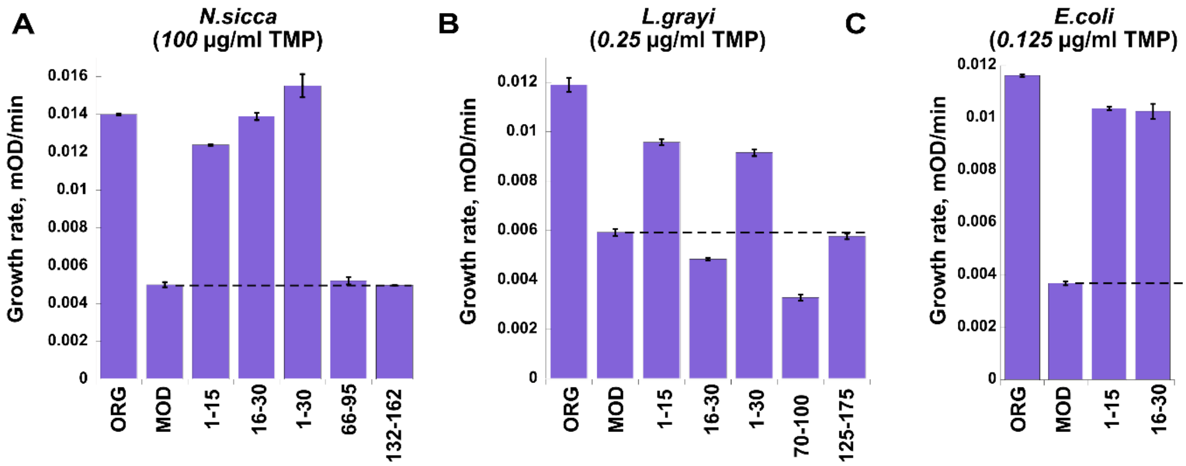


**Fig S11.** Changes in growth rates of *E. coli* strains carrying modified xenologous and endogenous *folA* genes. Growth rates of strains carrying fully modified (MOD), original (ORG) and chimeric *folA* sequences from (*A*) *N. sicca*, (*B*) *l. grayi*, and (*C*) *E. coli* in the presence of sub-MIC TMP (concentration is shown in parenthesis). Modified codons composing *folA* sequences (MOD) were partially replaced back to original codons by exchanging the modified codons found within codons 1-15, codons 16-30, codons 1-30, middle 30 codons (codons 66-95 in *N. sicca folA*, 70-100 in *L. grayi folA*), last 30 codons (codons 132-162 in *N. sicca folA*, 125-175 in *L. grayi folA*). Dashed lines mark the growth of strains with fully MOD *folA*.

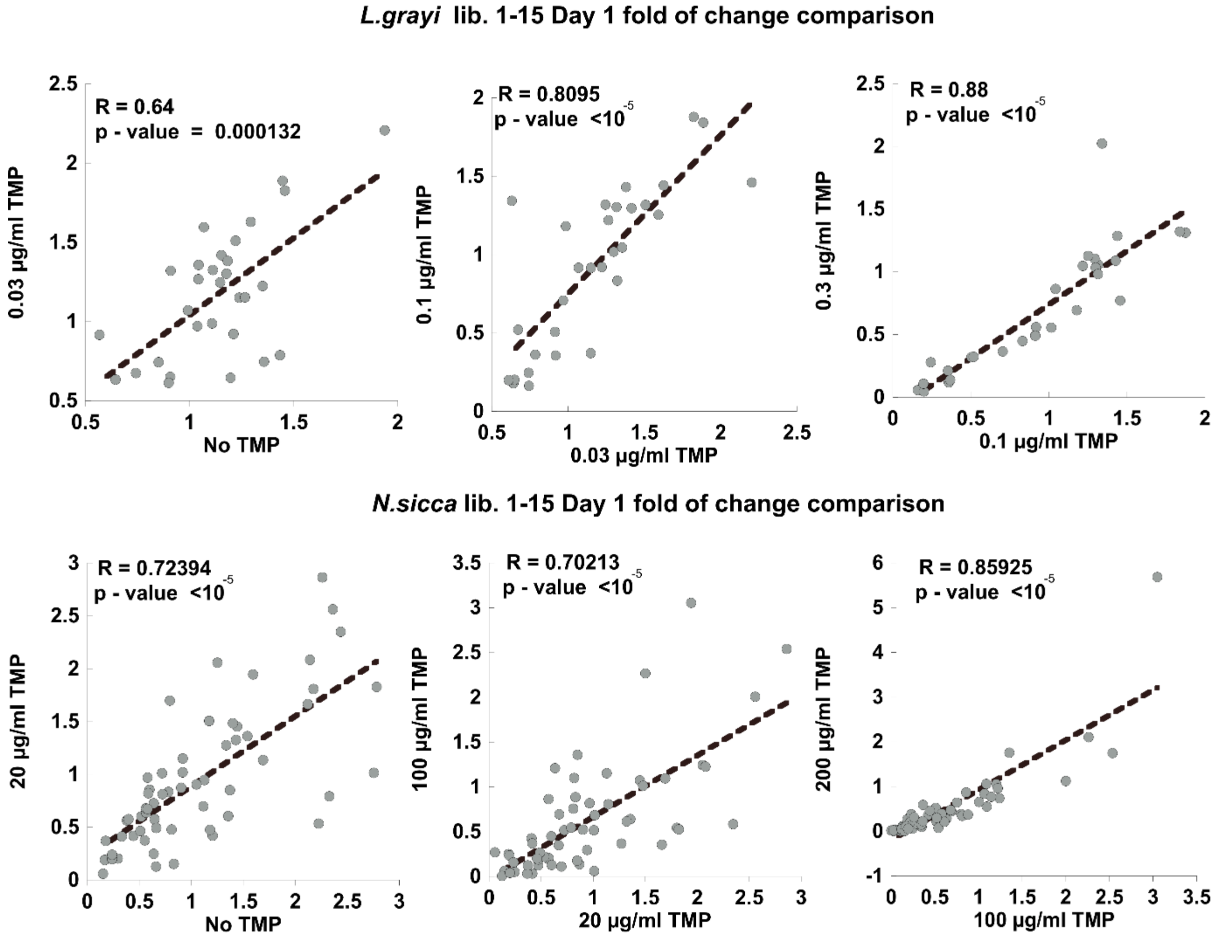


**Fig S12.** Fitness of variants (calculated as fold of change in normalized frequencies) correlates between populations subjected to comparable selection regimes. *Upper panel*, *L. grayi* lib. 1-15. *Lower panel*, *N. sicca* lib. 1-15. Pearson R and *p-values* are shown for each correlation. TMP concentrations correspond to selection conditions.

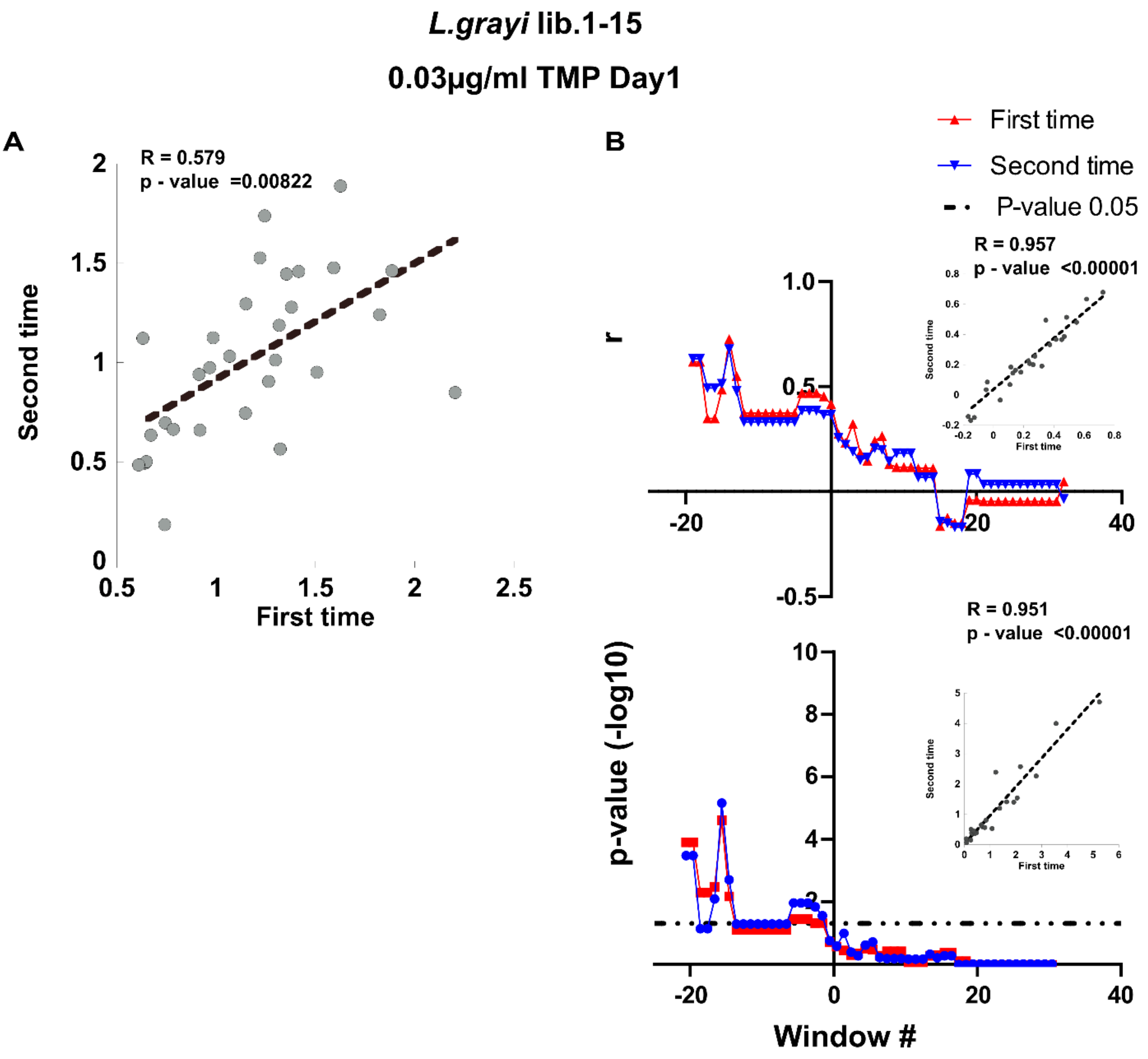


**Fig S13.** Biological repeat of selection on *L. grayi* lib. 1-15 at 0.03 μg/ml TMP. (*A*) Fold of change in the normalized frequencies of individual variants upon selection significantly correlates between both repeats (Pearson R and *p-values* are shown). (*B*) *P-values* for Spearman non-parametric test for association between fitness effects (fold of change in normalized frequencies of individual variants upon selection) and mRNA stability (calculated using a 30-nt long sliding window) in both biological repeats. *Lower panels.* Corresponding Spearman's correlation coefficients, *r*. Insets, Pearson correlations for the corresponding R and p-values between the biological repeats.

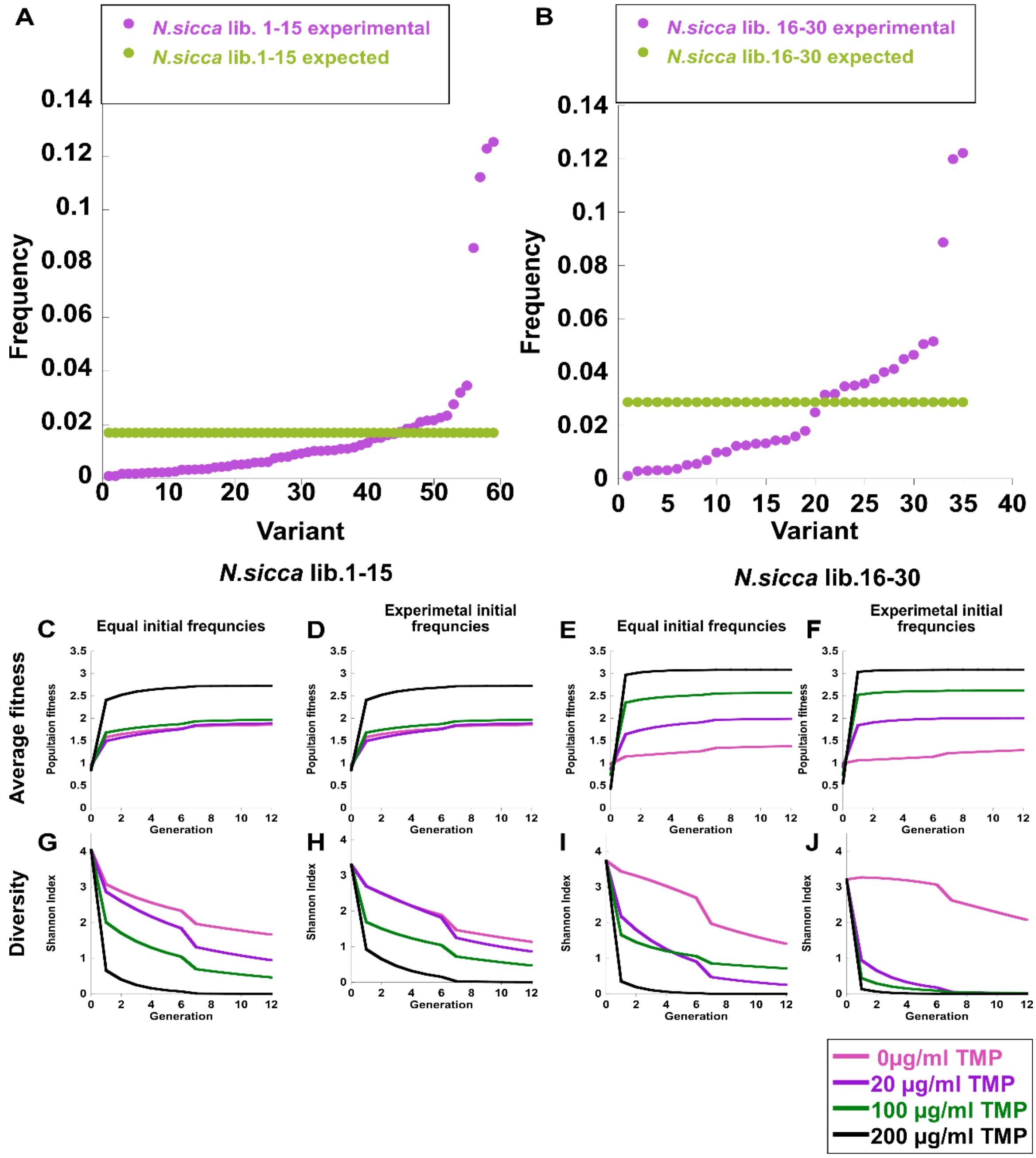


**Fig S14.** SodaPop simulations of population effects of selection on *N. sicca* libraries 1-15 and 16-30 run with equalized or experimentally defined initial library frequencies. (*A*, B) The expected (equalized, *green*) and experimentally determined (*pink*) initial frequencies of the library variants. (*C-J*) The change in the *N. sicca* lib. 1-15 and 16-30 population average fitness and Shannon diversity simulated with equalized (*C*, *G*, *E*, *I*) and experimentally determined (*D*, *F*, *H*, *G*) initial frequencies throughout 12 generation with 0, 20, 100, and 200 µg/ml TMP.

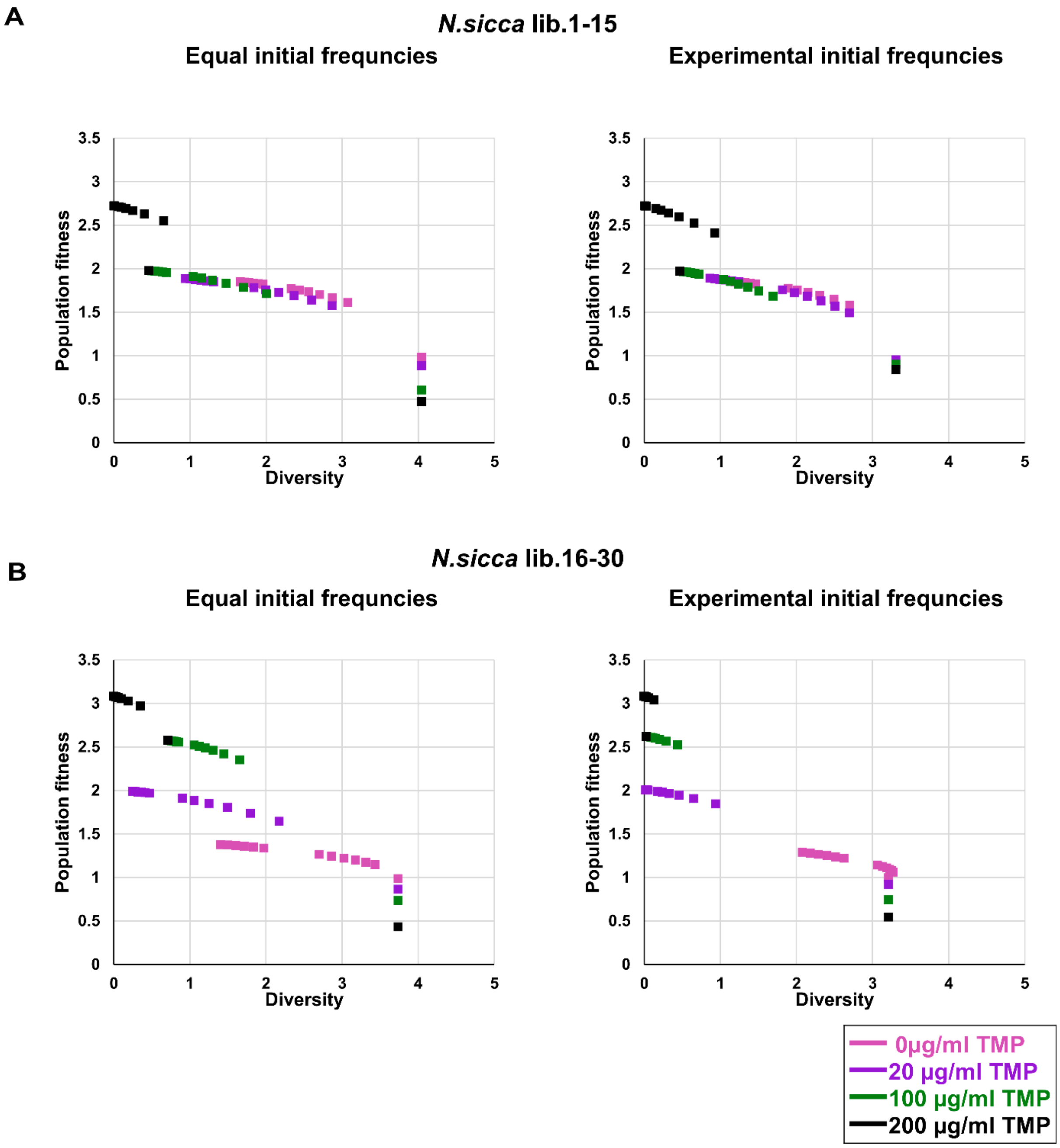


**Fig S15.** Correlation between the simulated population average fitness and Shannon diversity for *N. sicca* libraries 1-15 and 16-30. The simulated value for population fitness and diversity was calculated for 12 generations in 4 antibiotic concentrations (0, 20, 100, and 200 µgr/ml TMP). The starting point for each population is the highest diversity and lowest fitness (bottom right corner for each color). As the generations pass, the population average fitness increases and the population Shannon diversity decreases. The rate of this phenomenon corresponds to the slope for each condition (TMP concertation). In general, the higher the antibiotic concentration the faster the increase in the population fitness and drop in diversity. The average fitness of *N. sicca* lib. 1-15 reaches lower values that that of lib. 16-30. This is attributed to the increased clonal interference of the variants. This trend is similar when the initial frequencies are equal or biased, like in the real experiment.

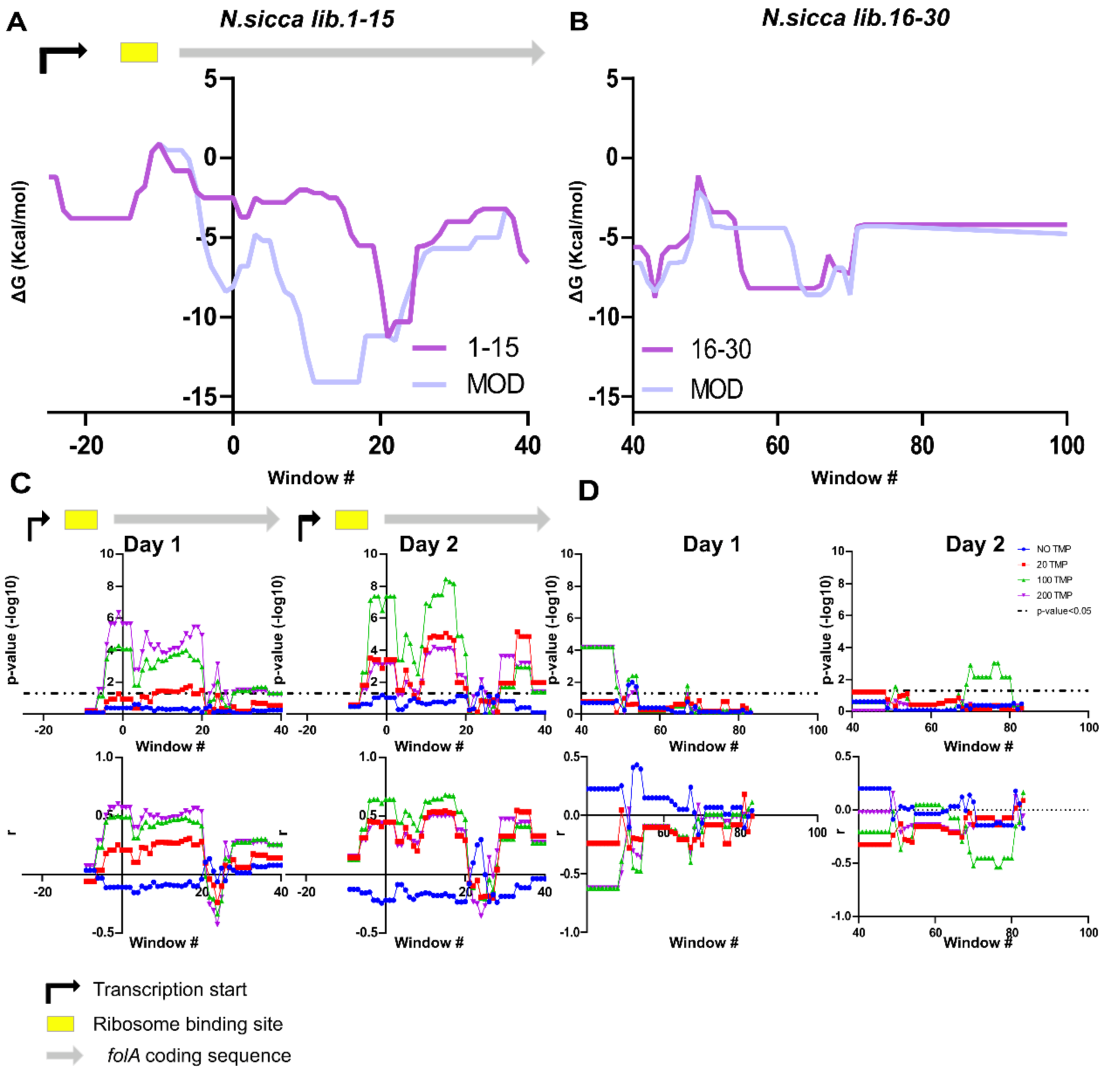


**Fig S16.** Correlation of fitness effects with mRNA stability in *N. sicca* libraries 1-15 and 16-30 after day1 and day2 of selection at a range of TMP concentrations. mRNA stability was calculated using a 30 nucleotide-long sliding window in steps of 1 nt starting (*A*) from nucleotide -25 and calculated for a modified (MOD) *N. sicca folA* sequence and sequence in which synonymous codons between codons (1-15) were replaced back to the original codons, (*B*) from nucleotide +40 and calculated for a modified (MOD) *N. sicca folA* sequence and sequence in which synonymous codons between codons (16-30) were replaced back to the original codons. (*C*, *D*) *Upper panels. P-values* for Spearman non-parametric test for association between fitness effects and mRNA stability. *Lower panels.* Corresponding Spearman's correlation coefficients, *r*.

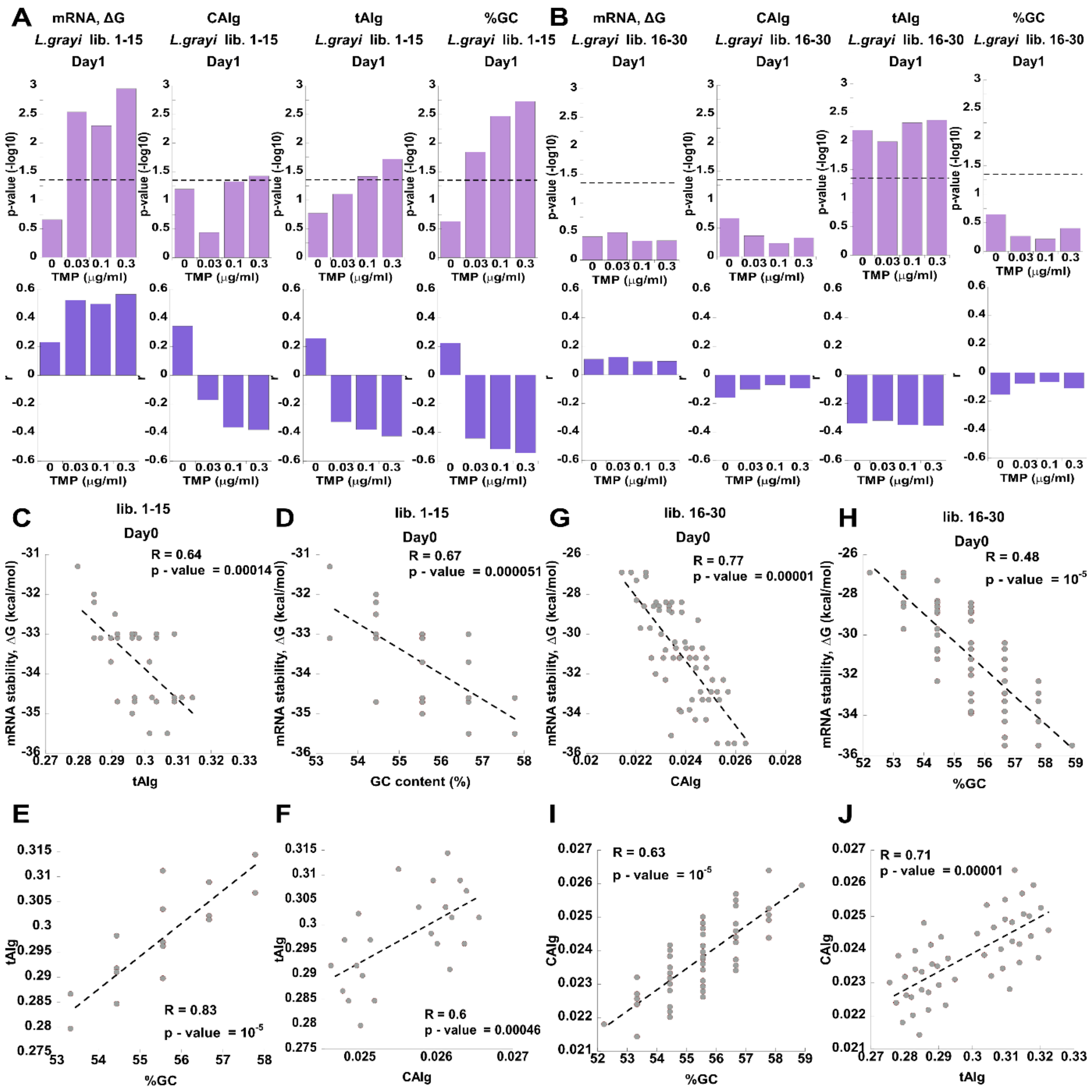


**Fig S17.** Correlation of fitness effects with *folA* sequence composition in *L. grayi* libraries 1-15 (*A*) and 16-30 (*B*) after day1 of selection at a range of TMP concentrations. (*A*, *B*) *Upper panels. P-values* for Spearman non-parametric test for association between fitness effects and mRNA stability (calculated for a single fragment spanning 114 nucleotides from nucleotide -25, the beginning of transcription, and up to nucleotide +90), codon optimality (CAIg, tAIg values), and GC content. *Lower panels.* Corresponding Spearman's correlation coefficients, *r*. (*C-J*). Pearson's correlations between mRNA stability, codon optimality (CAIg, tAIg), and GC content for variants comprising naïve libraries (day0). Pearson's p-values and R values are shown.

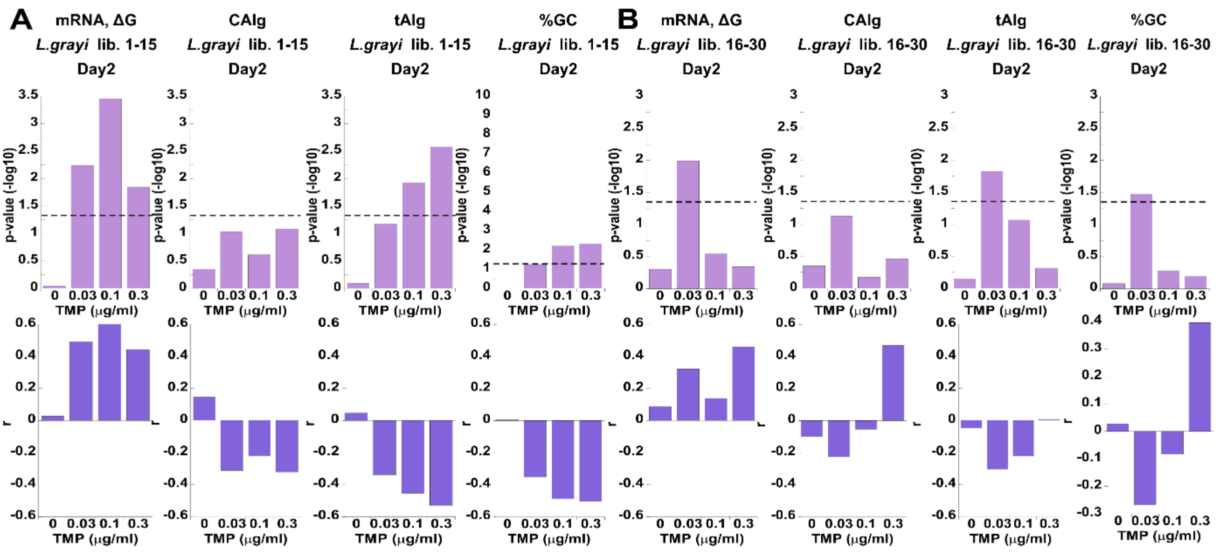


**Fig. S18.** Correlation of fitness effects with *folA* sequence composition in *L. grayi* libraries 1-15 (*A*) and 16-30 (*B*) after day2 of selection at a range of TMP concentrations. (*A*, *B*) *Upper panels. P-values* for Spearman non-parametric test for association between fitness effects and mRNA stability (calculated for a single fragment spanning 114 nucleotides from nucleotide -25, the beginning of transcription, and up to nucleotide +90), codon optimality (CAIg, tAIg values), and GC content. *Lower panels.* Corresponding Spearman's correlation coefficients, *r*.

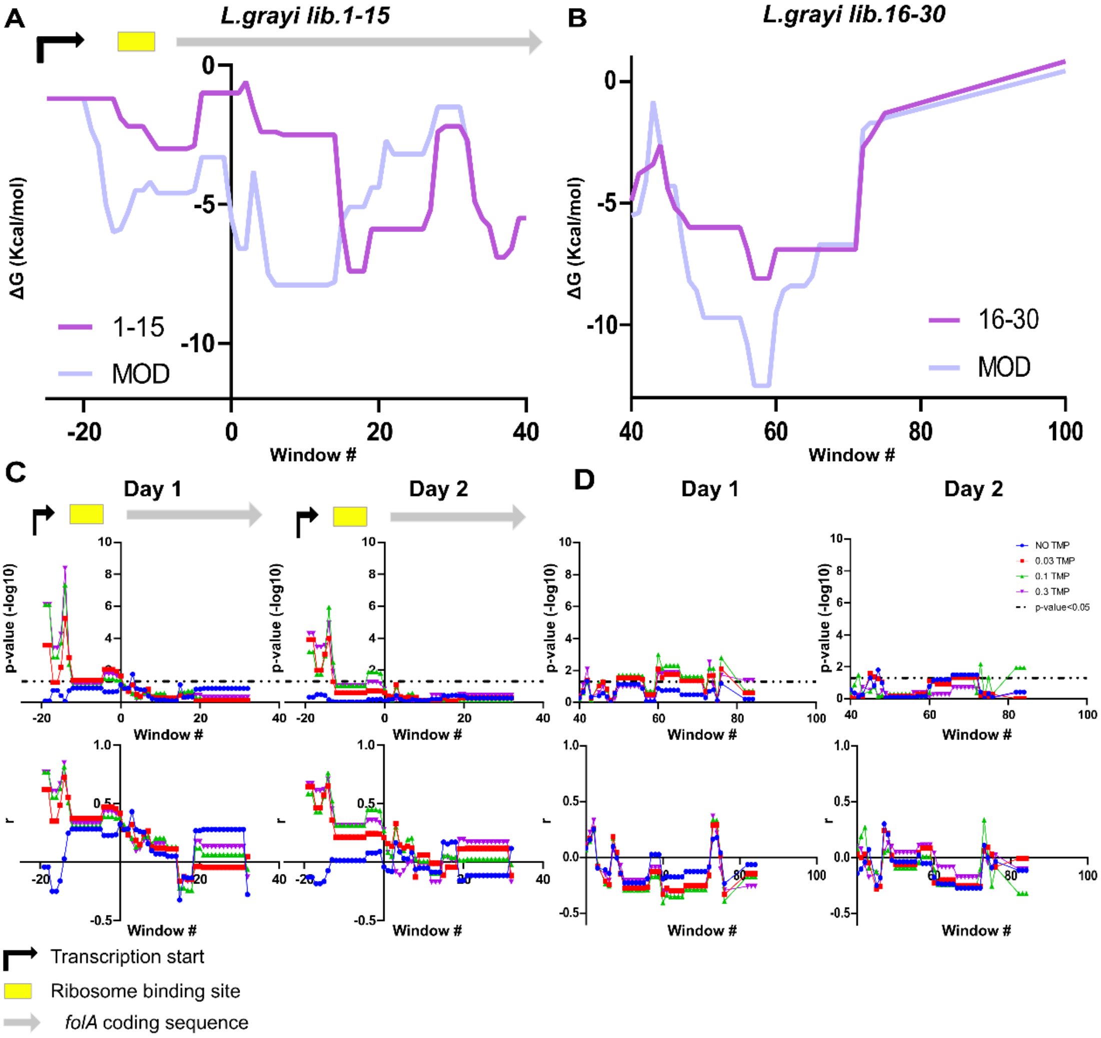


**Fig S19.** Correlation of fitness effects with mRNA stability in *L. grayi* libraries 1-15 and 16-30 after day1 and day2 of selection at a range of TMP concentrations. mRNA stability was calculated using a 30 nucleotide-long sliding window in steps of 1 nt starting (*A*) from nucleotide -25 and calculated for a modified (MOD) *L. grayi folA* sequence and sequence in which synonymous codons between codons (1-15) were replaced back to the original codons, (*B*) from nucleotide +40 and calculated for a modified (MOD) *N. sicca folA* sequence and sequence in which synonymous codons between codons (16-30) were replaced back to the original codons. (*C, D*) *Upper panels. P-values* for Spearman non-parametric test for association between fitness effects and mRNA stability. *Lower panels.* Corresponding Spearman's correlation coefficients, *r*.

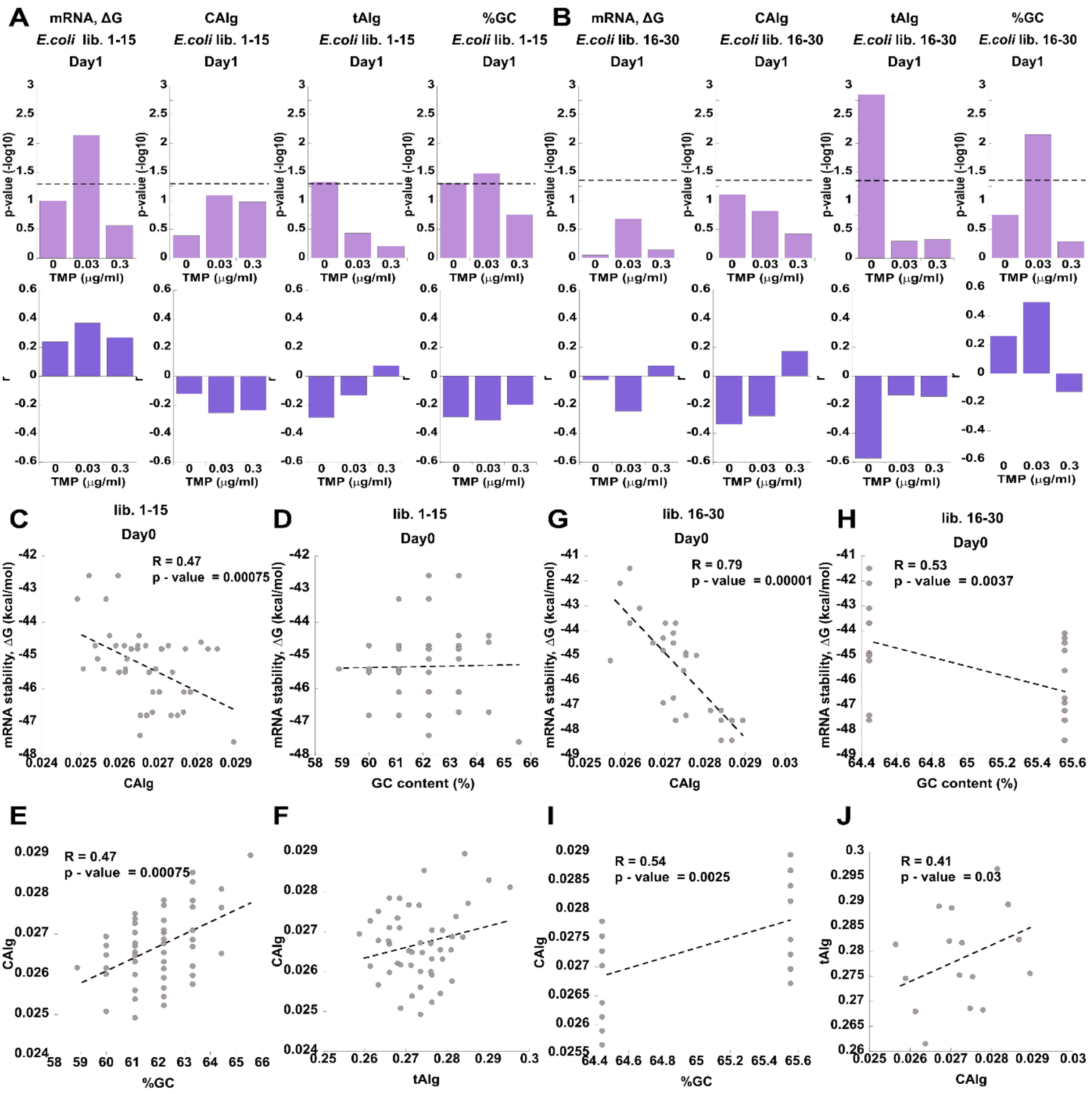


**Fig S20**. Correlation of fitness effects with *folA* sequence composition in *E. coli* libraries 1-15 (*A*) and 16-30 (*B*) after day1 of selection at a range of TMP concentrations. (*A, B*) *Upper panels. P-values* for Spearman non-parametric test for association between fitness effects and mRNA stability (calculated for a single fragment spanning 114 nucleotides from nucleotide -25, the beginning of transcription, and up to nucleotide +90), codon optimality (CAIg, tAIg values), and GC content. *Lower panels.* Corresponding Spearman's correlation coefficients, *r*. (*C-J*). Pearson's correlations between mRNA stability, codon optimality (CAIg, tAIg), and GC content for variants comprising naïve libraries (day0). Pearson's p-values and R values are shown.

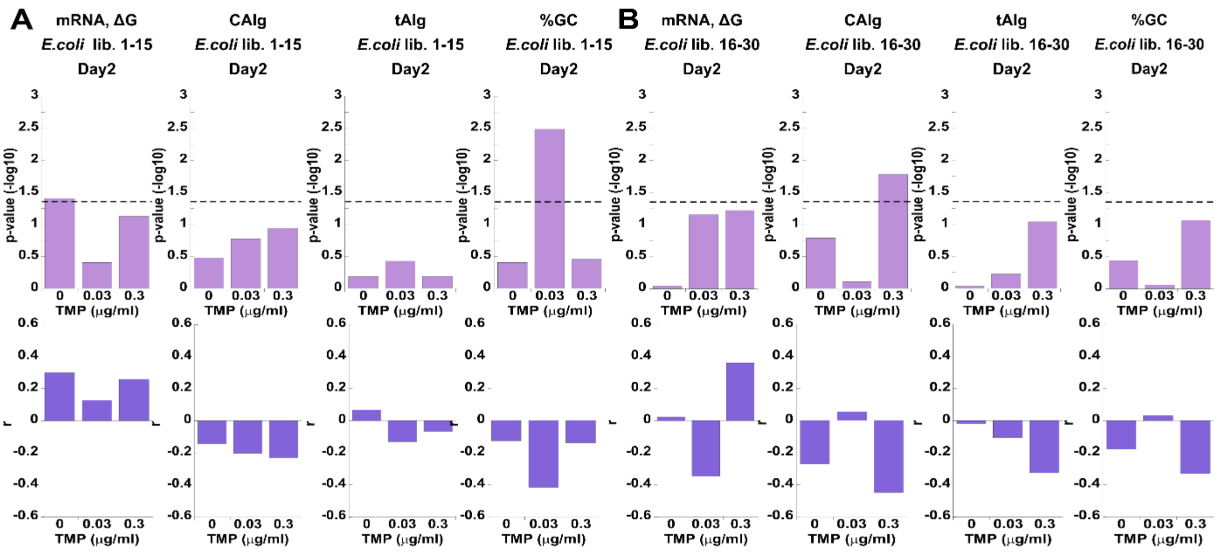


**Fig S21.** Correlation of fitness effects with *folA* sequence composition in *E. coli* libraries 1-15 (A) and 16-30 (B) after day2 of selection at a range of TMP concentrations. (*A*, *B*) *Upper panels. P-values* for Spearman non-parametric test for association between fitness effects and mRNA stability (calculated for a single fragment spanning 114 nucleotides from nucleotide -25, the beginning of transcription, and up to nucleotide +90), codon optimality (CAIg, tAIg values), and GC content. *Lower panels.* Corresponding Spearman's correlation coefficients, *r*.

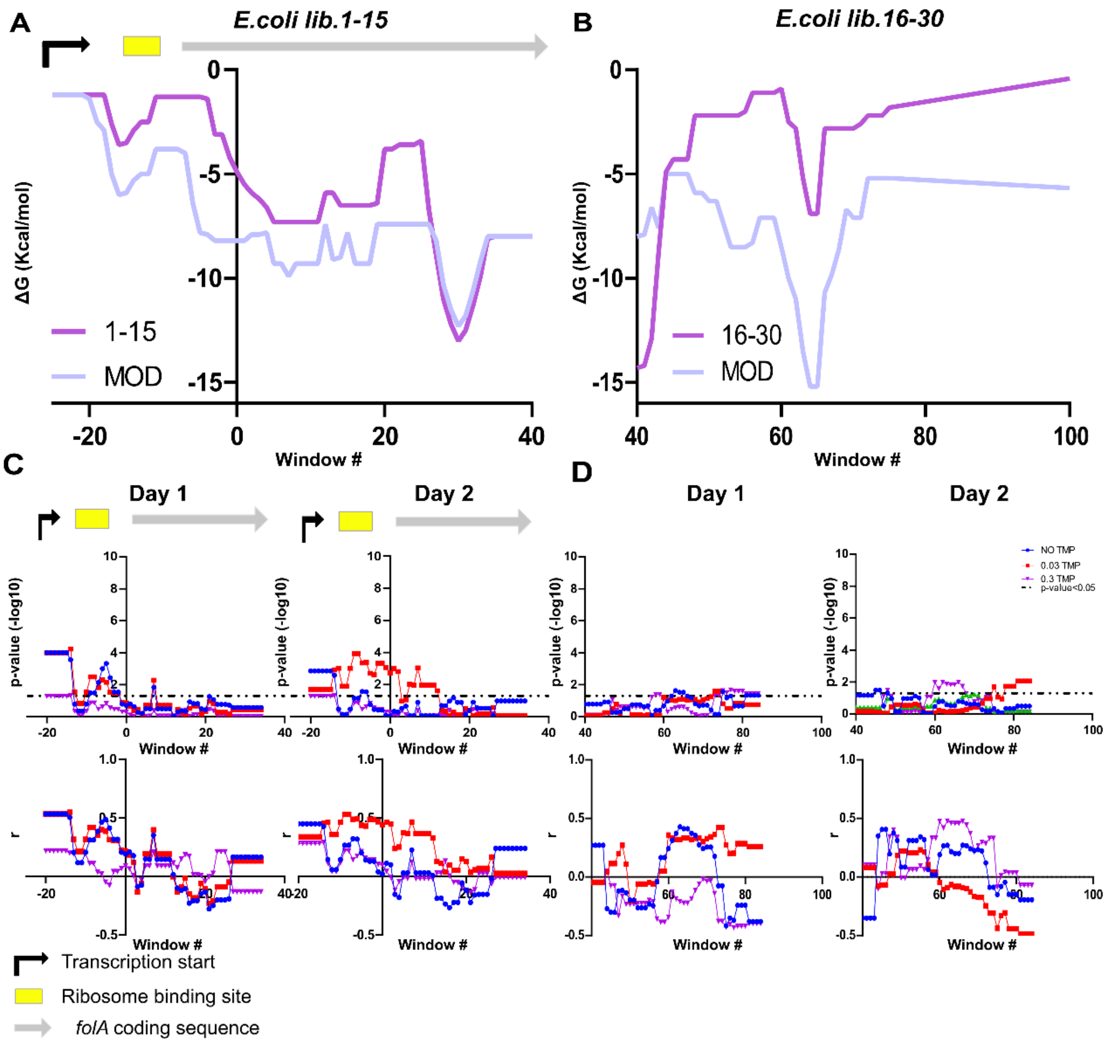


**Fig S22.** Correlation of fitness effects with mRNA stability in *E. coli.* libraries 1-15 and 16-30 after day1 and day2 of selection at a range of TMP concentrations. mRNA stability was calculated using a 30 nucleotide-long sliding window in steps of 1 nt starting (*A*) from nucleotide -25 and calculated for a modified (MOD) *E. coli folA* sequence and sequence in which synonymous codons between codons (1-15) were replaced back to the original codons, and (*B*) from from nucleotide +40 and calculated for a modified (MOD) *E. coli folA* sequence and sequence in which synonymous codons between codons (16-30) were replaced back to the original codons. (*C, D*) *Upper panels. P-values* for Spearman non-parametric test for association between fitness effects and mRNA stability. *Lower panels.* Corresponding Spearman's correlation coefficients, *r*.

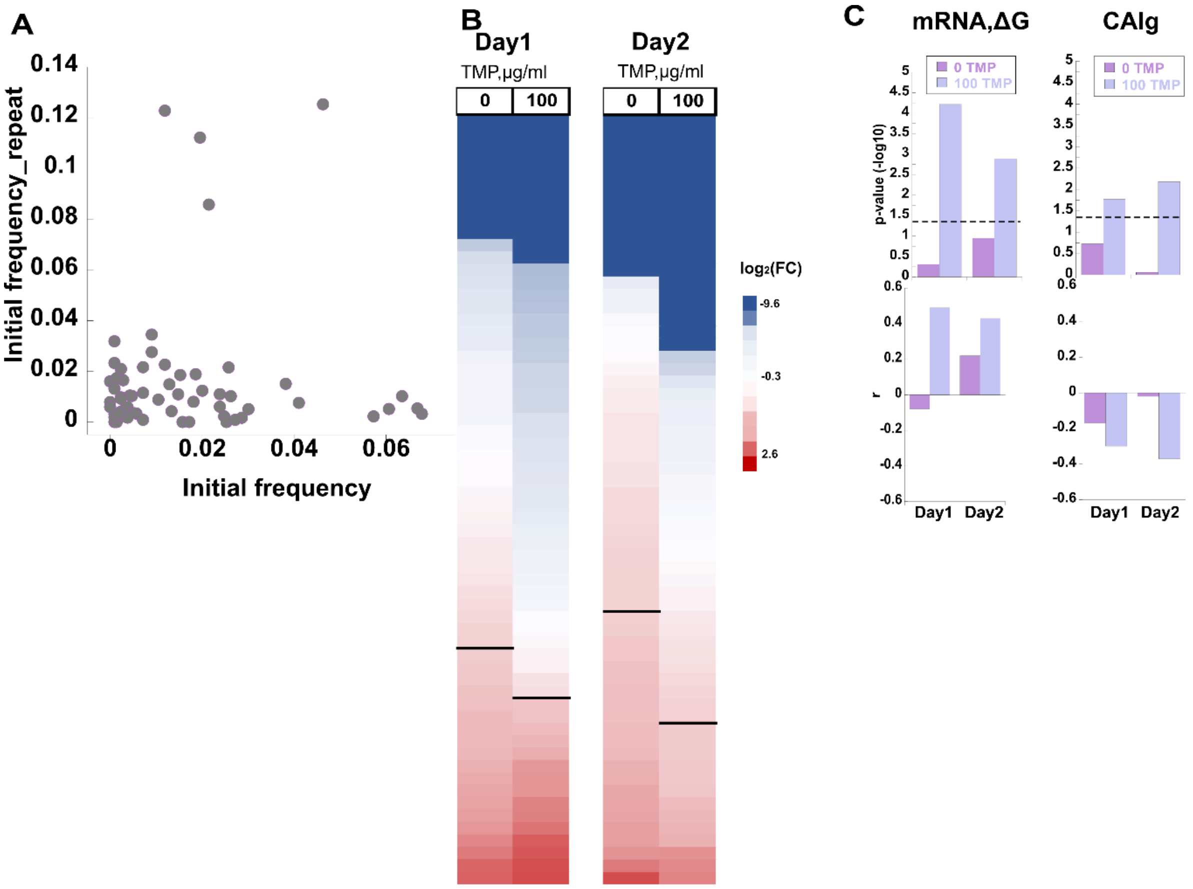


**Fig S23.** Repeat of selection on *N. sicca* lib. 1-15 after the perturbation in the initial variant frequencies. (*A*) Correlation between the initial frequencies prior to (Initial frequency) and after (Initial frequency_repeat) introduction of the perturbation. (*B*) $\log_2$(FC) of the individual library variants was sorted from lowest (*blue*) to highest (*red*) independently at each selection regime (no TMP and 100 µg/ml TMP) and presented as heat maps. (Note that variants at each row are not necessarily identical, as their fitness may change upon change in the selection regime). Black bars separate variants that were depleted upon selection ($\log_2$(FC) < 0) from those that were enriched ($\log_2$(FC) > 0). The heat map ruler indicates the fold change in the relative fitness of the individual variants. (*C*) *Upper panels. P-values* for Spearman non-parametric test for the association between the fitness effects and (*A*) mRNA stability (calculated for a single fragment spanning 114 nucleotides from nucleotide -25, the beginning of transcription, and up to nucleotide +90), and fitness effects and (*B*) codon optimality (CAIg). *Lower panels.* Corresponding Spearman's correlation coefficients, *r*.

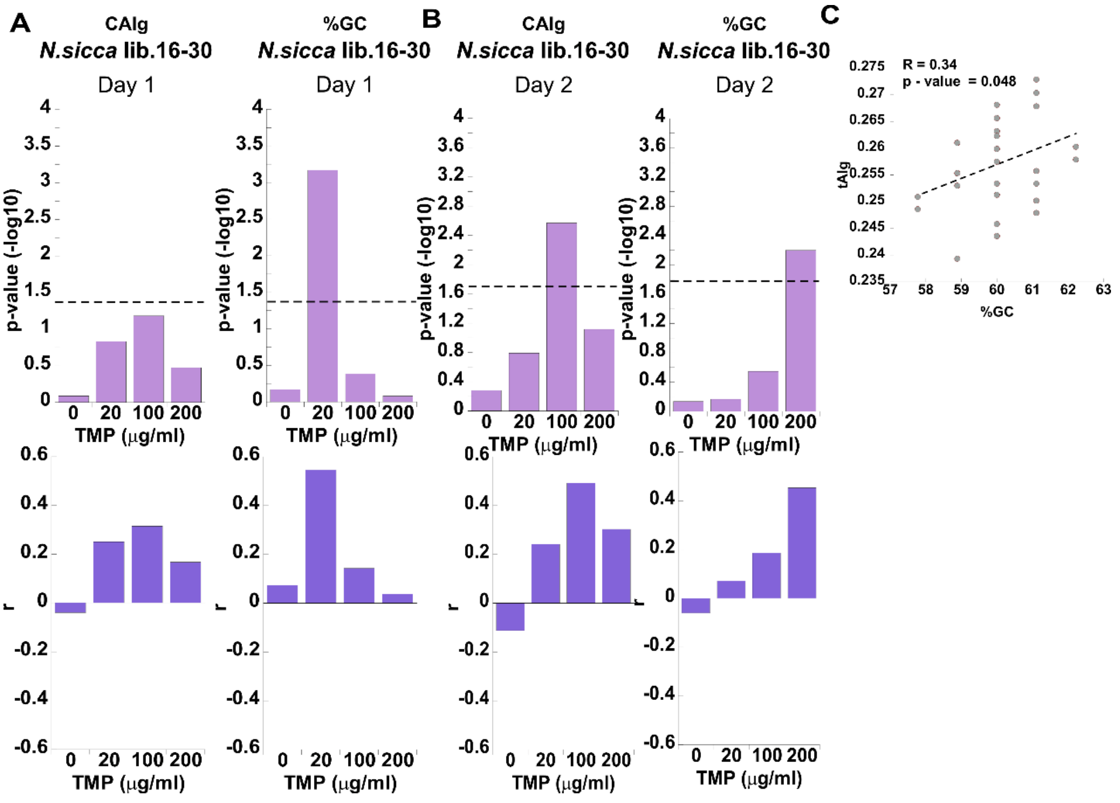


**Fig S24.** Correlation of fitness effects with *folA* sequence composition in *N. sicca* library 16-30 on day1 and day2 of selection at a range of TMP concentrations. (*A*, *B*) *Upper panels. P-values* for Spearman non-parametric test for association between fitness effects and codon optimality (CAIg values), and GC content. *Lower panels.* Corresponding Spearman's correlation coefficients, *r*. (*C*) Pearson's correlations between codon optimality (tAIg) and GC content for variants comprising the naïve libraries (day0). Pearson's p-value and R are shown.

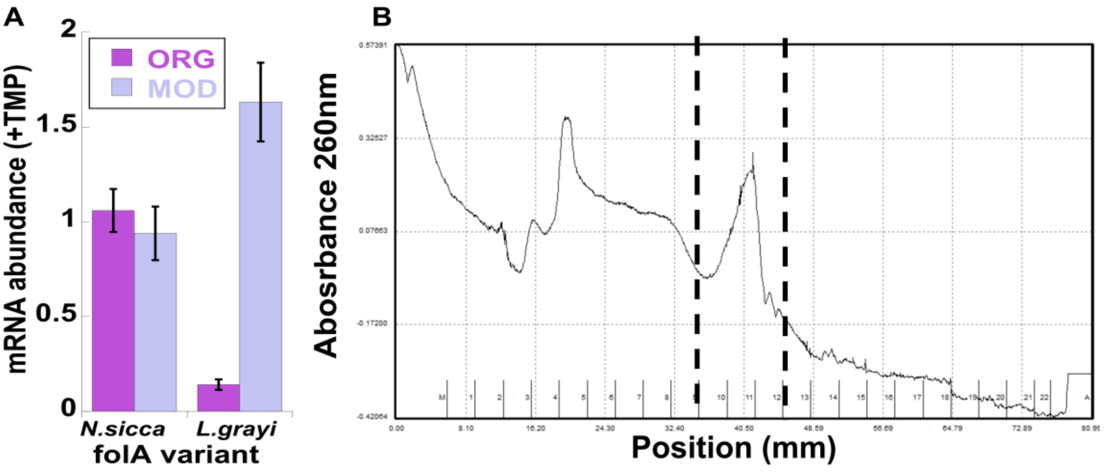


**Fig S25.** (*A*) The effect of codon usage on mRNA abundance in the presence of IC50 TMP concentrations. (*B*) A representative polysomal profile. The collected fraction is indicated within the dashed lines.

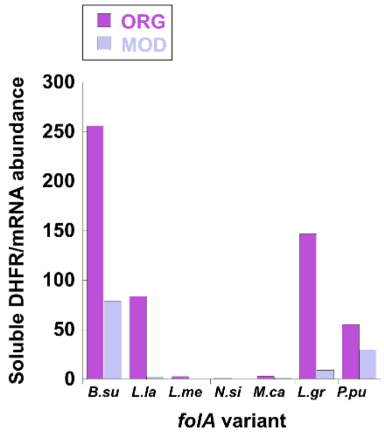


**Fig. S26.** Ratios between the DHFR intracellular protein abundance and *folA* mRNA abundance for each xenologous strain.
